# Emerging metallic nanotechnology platforms for cancer sensing and imaging

**DOI:** 10.1016/j.nantod.2026.103054

**Published:** 2026-04-22

**Authors:** Masoud Negahdary, William Skinner, Samuel Mabbott, Fay Nicolson

**Affiliations:** a Department of Biomedical Engineering, Texas A&M University, 101 Bizzel Street, College Station, TX 77843, USA; b Center for Remote Health Technologies & Systems, Texas A&M Engineering Experiment Station, 600 Discovery Drive, College Station, TX 77840-3006, USA; c Department of Radiation Oncology, Dana-Farber Cancer Institute, Harvard Medical School, Boston, MA 02215, USA

**Keywords:** Nanotechnology-based cancer diagnostics, Biosensing platforms, Molecular imaging nanoprobes, Point-of-care nanodevices, Electrochemical and optical sensing

## Abstract

Despite major advances, the management of cancer remains one of the greatest unresolved challenges in medicine and a leading cause of death worldwide. Thus, improved, early, and minimally invasive diagnostics are urgently demanded. In recent years, extraordinary progress in nanotechnology has radically changed this situation by providing unprecedented molecular-level insight into cancer cell mechanisms through highly sensitive biosensing platforms and multifunctional nanoprobes for advanced imaging. This review offers a unique perspective on emerging nanotechnology-based cancer diagnostics by examining the interconnected development of sensing and imaging technologies through the lens of nanomaterial physicochemical properties. In sensing, we highlight recent breakthroughs in electrochemical, optical, photoelectrochemical (PEC), enzymatic, surface-enhanced Raman scattering (SERS)-based, electroluminescence-PEC hybrid, molecular, fuel cell-powered, and nanocatalytic biosensing strategies. These approaches enable ultrasensitive and selective detection of a wide range of circulating biomarkers, including microRNAs (miRNAs), extracellular vesicles (EVs), exosomes, cell-free DNA, oncogenes, circular RNAs, circulating tumor cells (CTCs), telomerase activity, circulating proteins, methylation patterns, and tumor-associated carbohydrates. Beyond sensing, we also highlight significant advances in nanotechnology-based imaging, including magnetic resonance imaging (MRI), SERS, fluorescence, circular dichroism, and photoacoustic imaging. In these modalities, intelligent nanoprobes enhance specificity, resolution, and *in vivo* tissue imaging capabilities. Finally, we discuss the growing convergence of sensing and imaging nanotechnologies, the need for integrated theranostic platforms, translation into point-of-care devices, incorporation of artificial intelligence-driven analytics, and critical considerations related to nanobiomaterial safety. Collectively, these advances position nanotechnology as a transformative force in next-generation cancer diagnostics.

## Graphical Abstract

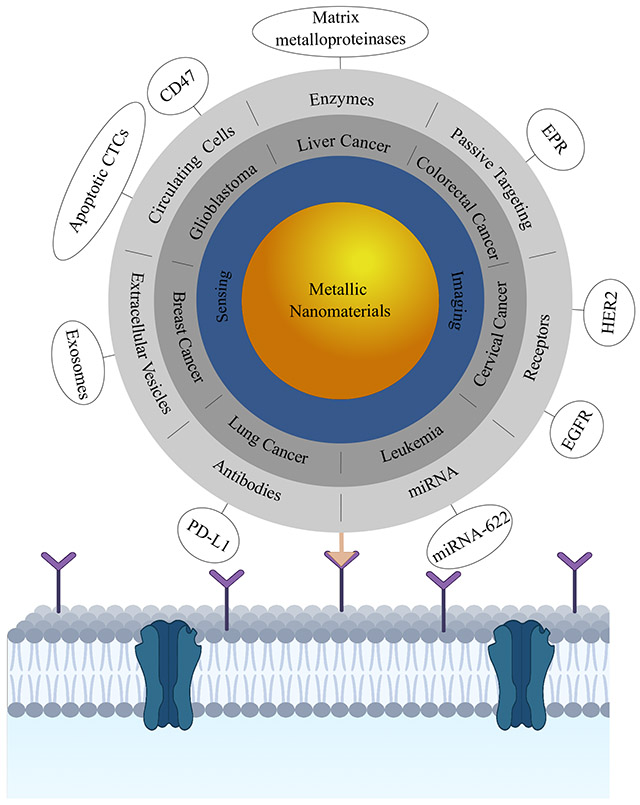

## Introduction

Cancer remains a major global health concern and one of the leading causes of death worldwide [1,2]. According to the latest available epidemiologic data, the global prevalence of cancer continues to rise, reflecting the combined effects of population aging, lifestyle, and environmental factors [1]. In the United States, approximately two million new cancer cases and more than 600,000 cancer-related deaths are expected in 2026 [3]. By 2050, the annual global incidence of cancer is expected to exceed 30 million cases, with mortality projected to surpass 16 million deaths if current diagnostic and treatment trends remain unchanged [2,4]. Despite significant advances in cancer management, five-year survival rates for several cancers, including pancreatic, stomach, liver, ovarian, and colorectal cancer (CRC), remain poor, particularly when diagnosed at advanced stages, underscoring the critical importance of early detection [5,6]. Survival rates depend heavily on the extent of disease progression, with drastic reductions observed as cancer progresses from the localized to the metastatic phase [1,7]. Current diagnostic practices rely primarily on imaging technologies, including positron emission tomography (PET), computed tomography (CT), and MRI, as well as histopathological and immunohistochemical analyses, blood-based biomarker assays, and tissue biopsies [8-10]. While these approaches provide invaluable anatomical, functional, metabolic, and morphological information, they also suffer from intrinsic limitations, including limited molecular specificity and sensitivity, restricted multiplexing capability, and spatial and temporal trade-offs. Moreover, cancers exhibit substantial molecular heterogeneity, thus complicating diagnosis, as no single biomarker can capture the full spectrum of tumor phenotypes [11]. Furthermore, tumors shed circulating biomarkers, including EVs, miRNAs, circulating tumor DNA (ctDNA), oncogenic proteins, and telomerase-associated nucleic acids, often at very early stages of disease and, in some cases, even before detection by conventional imaging modalities [12,13]. The diagnostic and prognostic value of such biomolecular patterns is immense, yet these patterns remain widely untapped in mainstream clinical practice due to the detection challenges posed by their low concentrations and instability [14,15].

In this context, nanotechnology has proven to be a revolutionary approach that can overcome these limitations and transform how cancer is detected and visualized [16-18]. The properties of nano-engineered materials include excellent physicochemical characteristics such as high surface-to-volume ratios, quantum properties, and plasmonic effects [19,20]. These properties have accelerated the creation of a new generation of cancer biosensors for blood, urine, and tissue liquid biopsies [21,22]. The various types of cancer biosensors include electrochemical, optical, photoelectrochemical, electrochemiluminescence (ECL), SERS, aptamer- and antibody-based, DNA nanostructure-assisted, and enzyme-powered sensors, and are known for their excellent analytical sensitivity, capable of detecting cancer at the attomolar scale [23-32]. In parallel, imaging has advanced substantially through the development of nanomaterial-based contrast agents and probes [33]. Although conventional radiologic imaging modalities are highly effective for clinical detection and staging, they are primarily anatomical and often lack molecular specificity. This limitation has been addressed through nanomaterial-based imaging strategies, including plasmonic SERS-based nanoprobes, magnetic nanoparticles (NPs) for MRI contrast enhancement, fluorophore-based quantum dots, upconversion NPs, and hybrid NPs [34-39]. These molecular imaging probes enable detailed imaging of the tumor microenvironment, quantification of enzyme activity, detection of proteolytic processes, visualization of surface receptor overexpression, and precise delineation of tumor boundaries with high resolution [40-42].

**Fig. 1.**
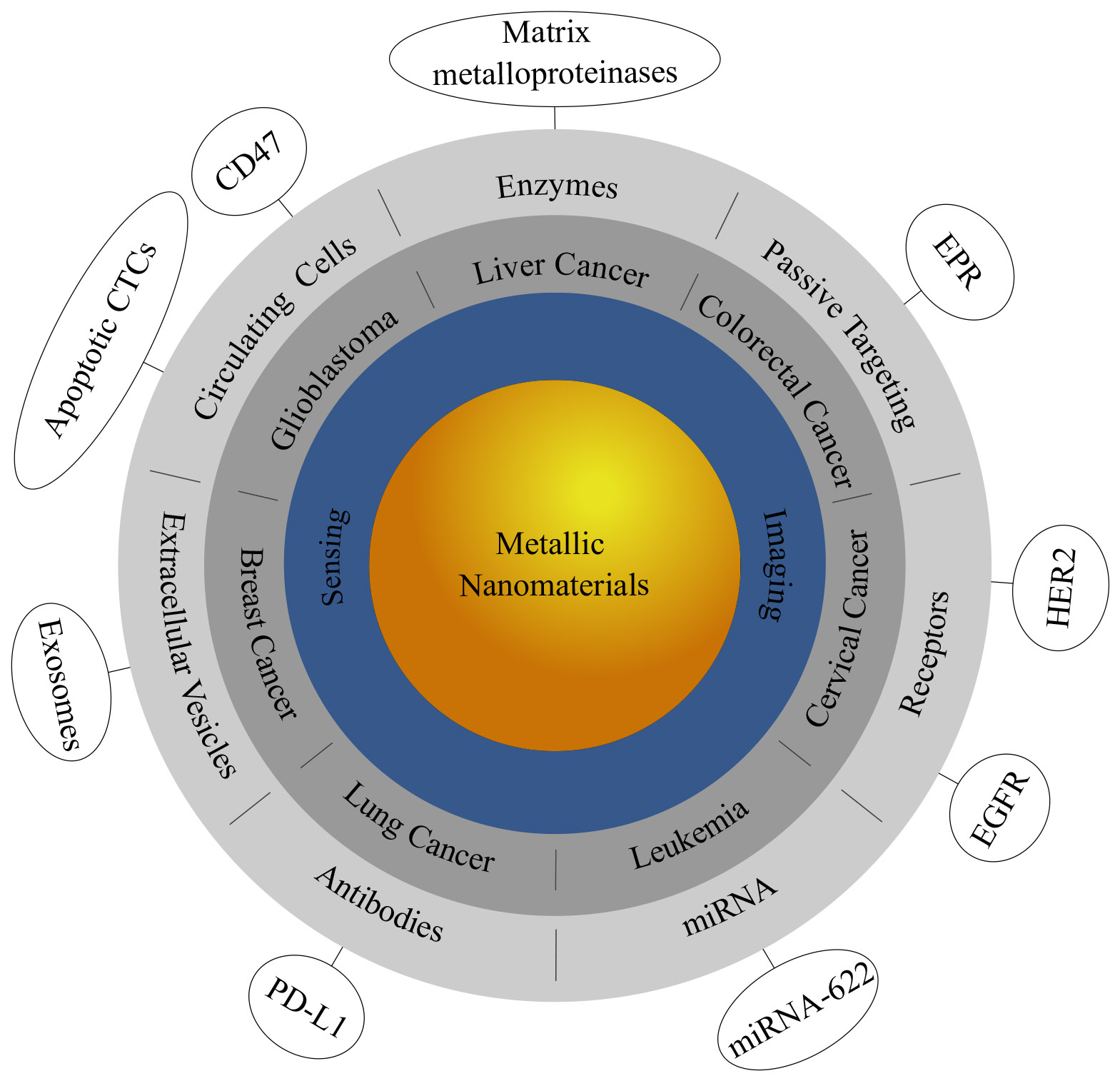
Schematic overview of metallic nanomaterials as a unifying platform for cancer sensing and imaging.

While several reviews have discussed nanotechnology in cancer diagnosis, most existing work focuses on either a single cancer type or a single technological aspect, such as metallic NPs or imaging modalities. For instance, previous studies have primarily examined metallic nanomaterials in general cancer therapy and diagnosis [43], metal nanoparticle applications specifically in lung cancer [44], or nanotechnology-based biomedical devices for oncology [45]. Other reports have concentrated mainly on imaging technologies such as nuclear or optical imaging modalities [33], or on early detection strategies using nanoparticle-enabled diagnostics [36,46]. In contrast, the present review provides an up-to-date, integrative perspective by systematically reviewing both nanotechnology-enabled sensing platforms and imaging techniques for cancer diagnostics. Importantly, this review examines multiple cancer types, allowing broader comparison of diagnostic strategies across oncology. By simultaneously analyzing biosensing approaches (electrochemical, optical, SERS, and molecular platforms) and advanced imaging modalities (MRI, fluorescence, photoacoustic, and multimodal imaging), this work highlights the convergence of sensing and imaging technologies and their potential for next-generation cancer diagnostics. Accordingly, this review provides a comprehensive and up-to-date framework for understanding nanotechnology-driven strategies for cancer detection. The review is organized into two main sections: Section 2 focuses on nanotechnology platforms for cancer sensing, while Section 3 reviews nanomaterial-based imaging approaches, highlighting their capabilities, limitations, and opportunities for integration. Emerging trends, including liquid biopsy integration, image-guided sensing, theranostics, and data-driven analysis, are discussed, and the review concludes with perspectives on future directions in convergent sensing and imaging technologies for cancer diagnostics. Fig. 1 presents a graphical summary of the conceptual basis, focus, and directional flow of this review.

## Recent innovative nanotechnologies for cancer diagnosis

### Major classes of cancer biomarkers and their biological origin

Cancer biomarkers represent measurable biological molecules that reflect tumor initiation, progression, or therapeutic response and are central to modern precision oncology and early diagnostic strategies. These biomarkers originate from diverse biological processes within the tumor microenvironment and can be broadly categorized into circulating nucleic acids, extracellular vesicle-associated molecules, circulating tumor cells, and tumor-derived proteins or metabolites. Many of these biomarker classes are released into biological fluids, including blood, urine, saliva, and cerebrospinal fluid, forming the basis of liquid biopsy approaches, which offer minimally invasive alternatives to traditional tissue biopsies for cancer detection and monitoring. Recent studies emphasize that liquid biopsies commonly contain ctDNA, CTCs, EVs, miRNAs, and other circulating RNAs that collectively reflect the molecular status of the tumor and its microenvironment [8]. Because these biomolecules can appear in circulation even during early tumor development, they have become key targets for next-generation diagnostic technologies. Among nucleic acid biomarkers, miRNAs constitute one of the most extensively studied classes. MiRNAs are small non-coding RNAs (approximately 19–25 nucleotides) that regulate gene expression at the post-transcriptional level and are frequently dysregulated during oncogenesis. Aberrant miRNA expression influences multiple cancer-related pathways, including cell proliferation, apoptosis, angiogenesis, and immune evasion. Importantly, tumor-associated miRNAs are highly stable in biofluids because they are either protein-bound or encapsulated within extracellular vesicles, making them particularly attractive candidates for non-invasive cancer diagnostics and prognostic monitoring [47]. Numerous studies have demonstrated that specific miRNA signatures can distinguish cancer patients from healthy individuals and may correlate with tumor stage, metastasis, and treatment response. A closely related biomarker class involves EVs and exosomes, nanoscale membrane-bound particles secreted by tumor and stromal cells. EVs carry complex molecular cargo, including proteins, lipids, mRNA, miRNA, and DNA fragments, that reflect the molecular composition of the parent tumor cell. Because EVs circulate widely in body fluids and protect their cargo from enzymatic degradation, they function as stable carriers of tumor-derived molecular information. Emerging evidence indicates that EV-associated nucleic acids and proteins can provide a real-time snapshot of tumor biology and are increasingly recognized as powerful diagnostic and prognostic biomarkers in multiple cancer types [48]. Recent clinical studies further demonstrate that exosome-derived miRNA signatures can achieve high diagnostic accuracy for early cancer detection in blood-based liquid biopsy assays [49]. Another critical biomarker category comprises CTCs. These intact malignant cells detach from primary tumors or metastatic lesions and enter the bloodstream during tumor dissemination. Although CTCs are extremely rare, often fewer than one cell per billion blood cells, they provide valuable biological information about tumor heterogeneity, metastatic potential, and therapeutic resistance. Clinically, enumeration and molecular profiling of CTCs have been associated with disease progression and prognosis across several cancers, including breast, lung, and colorectal cancers. Their detection, therefore, offers an opportunity not only for early diagnosis but also for real-time monitoring of tumor evolution. In addition to cellular biomarkers, tumors release fragmented nucleic acids such as cell-free DNA and ctDNA into the bloodstream through apoptosis, necrosis, and active secretion. CtDNA carries tumor-specific genetic alterations, including mutations, copy number variations, and methylation signatures that mirror the genomic landscape of the primary tumor. Because ctDNA can be detected even at very low concentrations during early disease stages, it has emerged as a powerful biomarker for early detection, molecular stratification, and monitoring of minimal residual disease in oncology. These biomarker classes, miRNAs, extracellular vesicles, circulating tumor cells, and circulating tumor DNA, represent complementary sources of molecular information about tumor biology. Their detection in liquid biopsies provides a non-invasive window into cancer development and progression. However, many of these biomarkers occur at extremely low concentrations and are present in complex biological matrices, posing significant analytical challenges. Consequently, highly sensitive analytical technologies are required to detect and quantify them reliably. In recent years, nanotechnology-enabled sensing platforms have emerged as particularly promising solutions, offering ultra-sensitive detection capabilities and multiplexed analysis of cancer biomarkers. The following sections, therefore, examine how nanomaterial-based biosensing and imaging strategies have been developed to detect these biomarker classes with improved sensitivity, specificity, and clinical applicability.

### Platforms developed for the diagnosis of breast cancer

Breast cancer remains one of the most prevalent malignancies worldwide and represents a major public health challenge. According to the American Cancer Society, breast cancer is the most commonly diagnosed cancer among women in the United States, with more than 310,000 new invasive cases expected annually, highlighting its substantial disease burden. Early detection is critically important because identifying tumors at earlier stages significantly improves treatment outcomes, survival rates, and the possibility of less aggressive therapies. Advances in screening technologies, such as mammography and emerging AI-assisted imaging approaches, have demonstrated that early detection enables earlier diagnosis and contributes to meaningful reductions in breast cancer mortality. Consequently, improving access to screening programs and diagnostic technologies remains a central strategy for reducing the global impact of breast cancer and enhancing patient prognosis [50,51].

Tumor cell-derived EVs have emerged as promising noninvasive biomarkers due to their elevated abundance in biological fluids and their ability to carry tumor-specific proteins, lipids, and nucleic acids. Importantly, EVs reflect the molecular composition and functional state of their parent tumor cells, enabling real-time insights into tumor progression, heterogeneity, and treatment response [52,53]. In one study, an electrochemical aptasensor was developed for the detection of breast cancer cell-derived EVs in human serum samples [54]. The sensing platform was equipped with spherical core-shell magnetic NPs and polydopamine (PDA) (Fe_3_O_4_@PDA, ~500 nm), which served as the capture probe due to their magnetism, biocompatibility, and abundant surface functional groups. The presence of PDA provided reactive quinone and catechol groups, facilitating the easy immobilization of aptamers for the specific recognition of EVs’ surface proteins. Aptamer-conjugated Fe_3_O_4_@PDA NPs were utilized to capture EVs from serum matrices, followed by electrochemical detection on an indium tin oxide (ITO) electrode. The detection mechanism was based on a sandwich-type format in which the aptamer-functionalized magnetic NPs trapped breast cancer EVs, which were then magnetically transported to the ITO electrode surface for electrochemical measurement. Differential pulse voltammetry (DPV) was used to convert the presence of EVs into detectable current responses. The magnetic capture method not only increased target enrichment and separation from nonspecific elements but also improved the assay’s reproducibility and sensitivity. The aptasensor exhibited a wide linear detection range from 700 to 1 × 10^6^ particles mL^−1^ and a very low limit of detection (LOD) of 220 particles mL^−1^. The assay confirmed high specificity, with minimal signal contributions from noncancerous EVs and other serum components.

Exosomes, a nanoscale subset of EVs, are considered promising biomarkers for cancer diagnosis because they carry proteins, nucleic acids, and other biomolecules that reflect the molecular characteristics of their parent tumor cells [55,56]. In breast cancer, exosomes represent a promising noninvasive diagnostic modality, as they carry tumor-specific molecular signatures. However, sensitive, reliable, and standardized detection remains challenging due to their nanoscale size, heterogeneity, and limitations of current analytical methods. To improve exosome detection, Zhang and colleagues developed a visual and fluorescence dual-mode platform (VFDMP) that utilized aptamer-based capture and signal amplification techniques [57]. The VFDMP applied Fe_3_O_4_ magnetic beads, supported with an epithelial cell adhesion molecule (EpCAM)-specific aptamer-ssDNA conjugate, was employed as a specific capture agent for MCF-7-derived breast cancer exosomes. After binding, the aptamer-activated single-stranded DNA triggered and stimulated a catalytic hairpin assembly (CHA) cycle, resulting in the release of silver ions from C-Ag^+^-C aggregates. Exosome capture was achieved by immobilizing aptamers on magnetic beads, followed by signal amplification via the CHA reaction without the need for enzymes, thereby reducing complexity and cost. Fluorescent reporters in the form of CdTe quantum dots were then used, driving a nanomaterial-assisted cation-exchange reaction in the presence of Ag^+^. This process suppressed both fluorescence and visual outputs in a concentration-dependent manner, thereby providing a dual readout-based method for exosome quantification. The concentration of exosomes directly controlled the amount of ssDNA released and, consequently, the level of fluorescence quenching during detection. Transmission electron microscopy, nanoparticle tracking analysis, and a Western blot confirmed the morphology and presence of biomarkers in the exosomes. VFDMP exhibited a remarkable detection range from 7.9 × 10^4^−2.2 × 10^6^ particles μL^−1^, with a fluorometric LOD as low as 1.1 particles μL^−1^ and a visual LOD of 300 particles mL^−1^. Clinical validation on human serum samples effectively distinguished control samples from cancer patients and further differentiated cancer stages (II and III) and subtypes (triple-negative, luminal B, and human epidermal growth factor receptor 2 (HER2)).

**Fig. 2.**
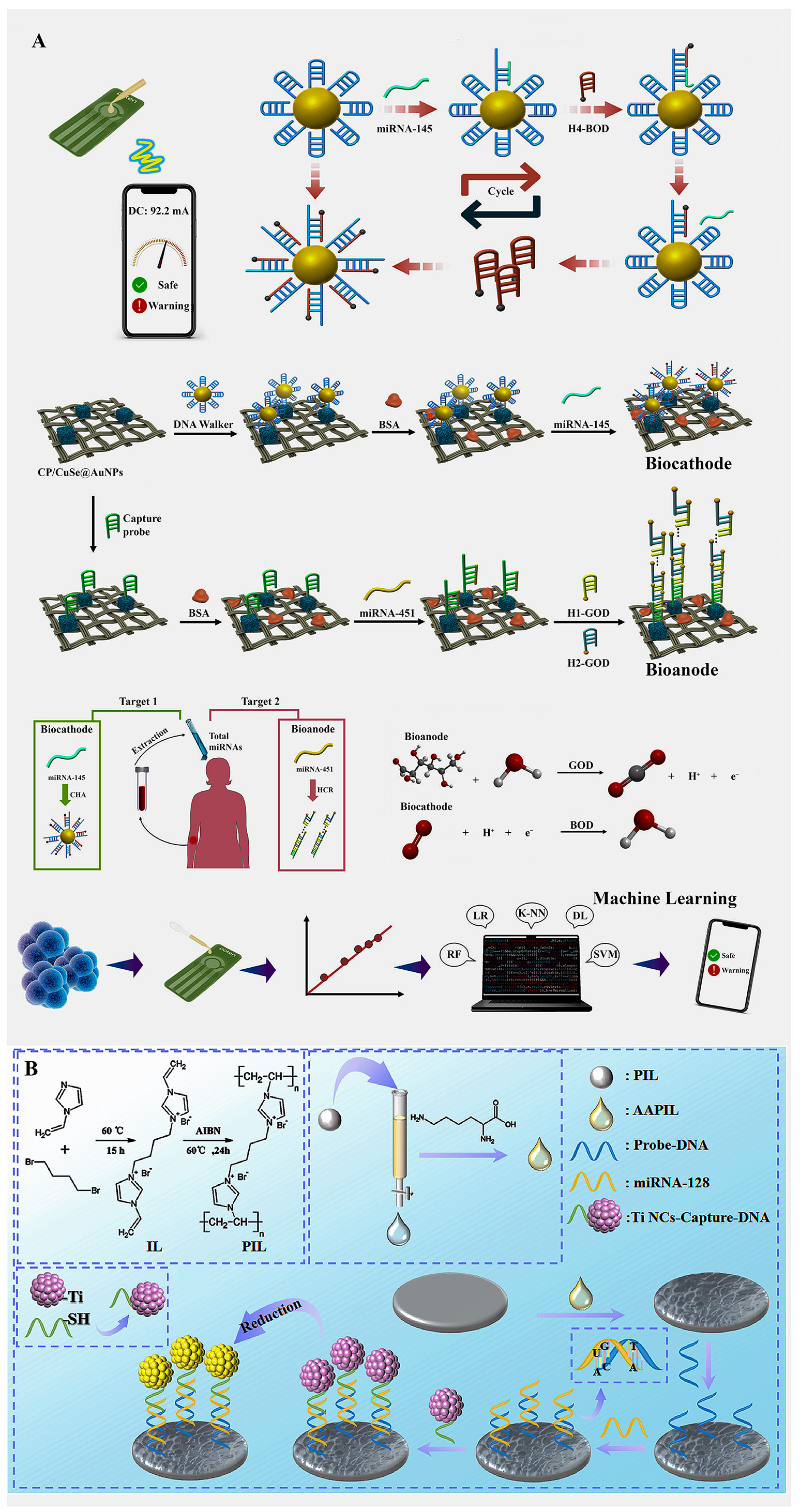
**A:** Overview of the machine learning-assisted multivariate sensing system for real-time monitoring of dopamine as a model neurochemical analyte; Reuse License Number: 6190541034514; [60]; and **B:** Schematic and performance summary of the AAPIL-based ECL biosensor for detection of miRNA-128; Reuse License Number: 6190541506326; [72].

Dysregulation of miR-145 and miR-451 has been reported across multiple cancer types, including colorectal, lung, pancreatic, ovarian, prostate, and brain cancers, where they are implicated in tumor progression, metabolic adaptation, and therapeutic resistance [58,59]. As key post-transcriptional regulators of gene expression, these miRNAs play central roles in controlling oncogenic signaling pathways, making their aberrant expression valuable for early diagnosis, prognosis, and treatment monitoring across diverse cancer types. Dual detection of the two miRNAs has been achieved using a bipolar self-powered electrochemical biosensor, developed to meet the requirements of a portable, ultrasensitive diagnostic tool for breast cancer detection in spiked serum samples [60]. Construction of the sensor involved creating a three-dimensional hollow cubic CuSe nanostructure decorated with uniformly dispersed gold NPs (AuNPs), which possessed high electrical conductivity, increased surface area, and abundant active sites for biomolecule attachment. Working electrode surfaces were modified with DNA assemblies that enabled enzyme-free amplification strategies, including a cascaded CHA cycle on a biocathode for identifying miRNA-145 and a hybridization chain reaction (HCR) process on a bioanode for identifying miRNA-451 (Fig. 2a). These reactions drove localized growth in DNA nanostructure, allowing for the immobilization of bilirubin oxidase on the cathode and glucose oxidase (GOD) on the anode, effectively integrating molecular-specific recognition with enzyme-catalytic reactions. Here, detection relied on a dual-enzyme biofuel cell-based mechanism, with glucose oxidation at the anode and oxygen reduction at the cathode, generating a detectable current; the target-triggered CHA and HCR reactions substantially boosted the electrochemical signal. The electrochemical assays provided quantitative determinations over a linear range from 1 to 100 fM, with LODs of 0.240 fM and 0.317 fM for miRNA-451 and miRNA-145, respectively. Integration into a commercial chip connected to a smartphone enabled real-time monitoring, and machine-learning algorithms improved predictive accuracy in multi-variable miRNA analyses.

CTCs play a central role in cancer metastasis and are clinically relevant biomarkers for cancer detection and prognosis, including in early-stage disease. In breast cancer, the presence and number of CTCs are associated with disease progression and metastatic risk [61,62]. However, because CTCs occur at extremely low frequencies in the bloodstream, the development of highly sensitive and specific biosensing strategies is essential for their reliable detection and for improving clinical outcomes. In a recent study, a dual-recognition ratiometric electrochemical biosensor was constructed for the ultra-sensitive detection of MCF-7 human breast cancer cells [63]. Two new nanomaterials, ferrocene-loaded porous organic cages (Fc@POCs) were synthesized and integrated with an electrodeposited gold film and utilized to immobilize EpCAM aptamers and methylene blue (MB)-encapsulated covalent organic frameworks (MB@COFs) modified with mucin 1 (MUC1) aptamers as a biosensing platform for the detection of MCF-7 human breast cancer cells. Fabrication of the sensor involved sequential modification of a GCE with Fc@POCs, electrodepositing an Au film, and immobilizing EpCAM aptamers, followed by incubation with target MCF-7 cells and labeling with MB@COFs-MUC1 aptamers. Selective capture of CTCs was achieved using a dual-aptamer configuration. The conductive substrate producing the internal reference signal was Fc@POCs, while the high dye-loading capabilities of MB@COFs were used for substantial signal amplification and ratiometric sensing, in which the ferrocene signal decreased upon binding to CTCs and the MB signal increased. This feature created an on-off complementary response that reduced background interference and improved the assay accuracy. Electrochemical square-wave voltammetry (SWV) was employed to monitor the ratio between the two signals (current of MB/current of Fc), which showed an excellent linear relationship with the concentration of MCF-7 cells from 10 to 1 × 10^7^ cells mL^−1^, achieving a LOD of 1 cell mL^−1^. Compared with the conventional single-signal biosensors, the presented ratiometric method effectively prevented false-positive errors.

### Platforms developed for the diagnosis of bladder cancer

Bladder cancer is one of the most common malignancies of the urinary system and represents a significant global health burden. It is characterized by high recurrence rates and substantial morbidity, making early diagnosis and continuous monitoring essential for effective disease management. According to the American Cancer Society, bladder cancer accounts for tens of thousands of new cases each year in the United States, with early-stage detection greatly improving survival outcomes and reducing treatment complexity. Advances in molecular diagnostics, imaging, and biomarker-based detection strategies have increasingly focused on enabling earlier and more accurate identification of bladder tumors. Such early detection approaches are critical for improving prognosis, guiding personalized therapeutic interventions, and reducing disease recurrence and mortality [64-66]. MiRNAs such as miRNA-183 and miRNA-155 have emerged as potential urinary biomarkers due to their increased expression in bladder cancer patients, their association with tumor stage, and their association with recurrence, providing a noninvasive pathway for diagnosis [67,68]. In work by Lin et al., a multiplex biosensing platform consisting of pH-responsive triplex DNA nanoswitches coupled with surface plasmon resonance (SPR) was fabricated to detect miRNA-183 and miRNA-155 simultaneously [69]. Two different triplex probes, switch A and switch B, were designed and engineered to show pH-induced structural changes. Immobilization of the mentioned probes was performed on a CM5 SPR chip, which was previously modified with the S9.6 antibodies (specific for the DNA/RNA hybrid), to ensure the specific attachment of the complexes between the probe and the miRNA. The streptavidin-AuNPs, attached via biotinylated reporter strands, served as signal-amplification labels that enhanced the SPR signal through plasmonic coupling at the sensor interface. The detection process was based on the pH-induced conformational change of the triplex DNA, while at pH 5.0, switch A was converted into a C-G·C+ triplex, enabling the release of the AuNPs-tagged reporters and, as such, the quantitation of miRNA-183. Also, at pH 8.3, switch B directed the T-A·T triplex, enabling the release of a second set of reporters that corresponded to the recognition of the miR-155. At each release event, the SPR reflectivity decreased sequentially, directly proportional to the target concentration, thus enabling both sequential and multiplexed detection in a single shot. The assay showed exceptional analytical sensitivity, with LODs of 0.57 pM for miR-183 and 0.83 pM for miR-155, and a dynamic range between 10 pM and 10 nM. Compared to single-target detection, the dual assay demonstrated high reproducibility with no significant cross-reactivity, even in the presence of cancer-related miRNAs. The platform also showed high recovery rates above 90% in artificial urine. Verification with clinical samples from bladder cancer patients and standard controls supported the assay’s diagnostic usability, as values strongly correlated with qRT-PCR results.

### Platforms developed for the diagnosis of CRC

CRC is among the leading causes of cancer-related morbidity and mortality worldwide and represents a major challenge for global healthcare systems. According to the American Cancer Society, CRC is one of the most commonly diagnosed cancers and remains a leading cause of cancer-related deaths in both men and women. The prognosis of CRC is strongly dependent on the stage at diagnosis, with early-stage detection significantly improving survival rates and treatment success. Consequently, the development of effective screening and early diagnostic strategies, including colonoscopy, fecal biomarker tests, and emerging molecular detection technologies, has become a critical priority for reducing disease burden and mortality. Improving early detection and surveillance strategies, therefore, plays a central role in CRC control and in optimizing patient outcomes. [70,71]. Among the molecular biomarkers associated with CRC, miRNA-128 is a significant target, as its downregulation is directly correlated with tumor proliferation, migration, and adverse prognosis [59]. In response to the limitations of traditional miRNA detection methods such as RT-qPCR and Northern blotting, which, despite high sensitivity and specificity, require complex workflows and specialized equipment, researchers developed an ECL biosensor based on valence-modulated reduced titanium nanoclusters (R-Ti NCs) and an amino acid–based polyionic liquid (AAPIL), enabling rapid detection with enhanced luminescence and improved fouling resistance [72]. The biosensor was fabricated by modifying a glassy carbon electrode (GCE) with a superhydrophilic AAPIL, which increased the electrochemically active surface area and imparted strong antifouling resistance by forming a stable hydration layer. Covalent immobilization of the probe DNA was carried out on the AAPIL-modified electrode, followed by hybridization with the target miRNA-128 and conjugation with Ti NC-capture DNA (Fig. 2b). An electrochemical reduction was conducted on the Ti NCs to enhance the luminescent efficiency, as the reduced clusters showed five times the ECL intensity compared to the unreduced Ti NCs. The AAPIL enabled fast electron transfer and possessed a wide electrochemical stability window, allowing stable and robust ECL emission upon detection. The detection process involved hybridization between the target miRNA-128 and the probe DNA, which immobilized Ti NCs near the surface of the electrode. These interacted with H_2_O_2_ as the co-reactant, generating reactive species that excited the nanoclusters and produced an ECL signal proportional to the target concentration. Furthermore, the antifouling characteristics imparted by the AAPIL modification enabled reliable measurements in complex biofluids, while stability testing confirmed retention of ~95% of the initial response over a five-day period. The biosensor possessed a remarkably low LOD of 0.17 fM and a broad dynamic range from 1 fM to 1 nM. The platform exhibited excellent reproducibility, with a RSD as low as 1.93% across multiple electrodes. High selectivity was also demonstrated, with minimal cross-reactivity observed toward non-target miRNAs, including miR-21, miR-205, miR-221, and miR-223–3p, which are commonly dysregulated in cancers such as breast, colorectal, lung, ovarian, and prostate cancers. This minimal cross-reactivity highlights the sensor’s ability to discriminate target miRNAs from closely related oncogenic miRNAs, a critical requirement for accurate cancer diagnosis in complex biological samples.

### Platforms developed for the diagnosis of liver cancer

Liver cancer, predominantly hepatocellular carcinoma (HCC), represents one of the most lethal malignancies worldwide due to its aggressive progression and frequent diagnosis at advanced stages. According to the American Cancer Society, liver cancer incidence and mortality have increased substantially in recent decades, making it a major global health concern. Early detection is particularly critical because patients diagnosed at early stages have significantly improved survival rates and may benefit from potentially curative treatments such as surgical resection, liver transplantation, or localized ablation therapies. However, many cases remain asymptomatic in early stages, highlighting the urgent need for improved screening strategies, sensitive biomarkers, and advanced diagnostic technologies to enable timely diagnosis and improve clinical outcomes [73,74].

Heat shock protein 70 (HSP70) is a stress-inducible molecular chaperone that plays a key role in tumor cell survival, proliferation, and resistance to apoptosis [75,76]. Its expression is elevated in approximately 70–80% of cases of HCC. Notably, HSP70 is significantly upregulated in early-stage HCC compared with precancerous or noncancerous liver tissues, highlighting its potential utility as a biomarker for early diagnosis and monitoring disease progression [77, 78]. To exploit the potential of HSP as a biomarker for the early detection of HCC-associated HSP70, an electrochemical sensor was developed featuring a framework made of a magnetic nanocomposite, Fe_3_O_4_@Au, and conjugated with a high-affinity HSP70 aptamer (Apt7) [79]. Spherical Fe_3_O_4_@AuNPs, with diameters from 200 to 350 nm, were synthesized through a chemical reduction method. Electrical conductivity was enhanced by applying a gold coating on the Fe_3_O_4_ particles, followed by Apt7 immobilization. This nanohybrid integrated the magnetic properties of Fe_3_O_4_, enabling facile attachment to a magnetic GCE, with the high conductivity of Au, which facilitated efficient electrochemical signal transduction. When human serum samples containing HSP70 were tested, the protein selectively bound to Apt7, which significantly increased the charge-transfer resistance (achieved by Electrochemical impedance spectroscopy (EIS)) due to the formation of a protein-aptamer complex. The developed aptasensor exhibited a linear detection range from 10 pg mL^−1^ to 200 ng mL^−1^, with an LOD of 0.525 pg mL^−1^ and an LOQ of 0.01 ng mL^−1^.

An electrochemical biosensor that integrated the specificity of lectinglycoprotein discrimination with the signal-enhancing capability of nanomaterials was developed for the detection of AFP, a valuable biomarker for HCC, the most common type of liver cancer [80]. The sensor platform used AgNPs conjugated with Lens culinaris agglutinin (LCA), a lectin with exceptional affinity for glycosylated forms of AFP. The spherical LCA-modified AgNPs functioned as an effective capture agent, a selective recognition component, and an electroactive tag, thereby enhancing both capture efficiency and signal transduction. Here, a GCE was employed for electrochemical measurements. Detection relied on a sandwich-type assay in which AFP in human serum samples specifically bound to LCA-functionalized AgNPs. This binding produced DPV-detectable current responses, which were amplified by the conductive properties of the AgNPs and the high affinity that LCA has for AFP glycoforms. Using lectin instead of antibodies reduced significant cross-reactivity and improved specificity for tumor-associated AFP, offering a notable advantage over traditional immunoassays. Optimizing the parameters, including incubation time, AgNPs concentration, and electro-preparation, further improved sensitivity and reproducibility. The biosensor possessed a linear detection range from 5 to 150 ng mL^−1^, with a LOD of 69 pg mL^−1^.

An electrochemical lateral flow immunoassay (e-LFIA) platform was also developed as a rapid and sensitive method for AFP detection, combining the ease of use of traditional lateral flow assays with a high-sensitivity electrochemical readout [81]. The sensor incorporated dendritic mesoporous silica nanoscaffolds (DMSNs) coated with Au NPs and a uniform silver nanoshell (DMSNs/AuNPs@Ag), providing a high surface area, good biocompatibility, and enhanced electrochemical activity to amplify the signal. These nanocomposites were transformed into an electrochemical immunoprobe by conjugating with secondary anti-AFP antibodies, while the primary anti-AFP antibodies were covalently linked to a nitrocellulose test membrane (Fig. 4E). Detection relied on a sandwich immunoassay format, where AFP antigens in the serum samples first attached to the DMSNs/AuNPs@Ag-antibody probes, and the resulting complexes were then captured at the test strip test line by immobilized antibodies. The silver shell enabled reliable readout via voltammetry, yielding clear DPV peaks proportional to AFP concentrations. Point-of-care electrochemical detection using screen-printed electrodes (SPEs) provided rapid results in less than 20 min. The platform could detect the analyte in a linear range up to 500 ng mL^−1^ with a low LOD of 0.85 ng mL^−1^, which is much lower than the clinical significance level of 10 ng mL^−1^. In addition, the biosensor was highly selective in the presence of interferences, including CEA, PSA, cancer antigen 125 (CA-125), and CA19–9. The stability tests demonstrated that the platform maintained stable performance after nine days of storage at 4 ^◦^C. Additionally, the recovery studies in human serum confirmed the accuracy, with percentages ranging from 92% to 105%.

### Platforms developed for the diagnosis of oral cancer

**Fig. 3.**
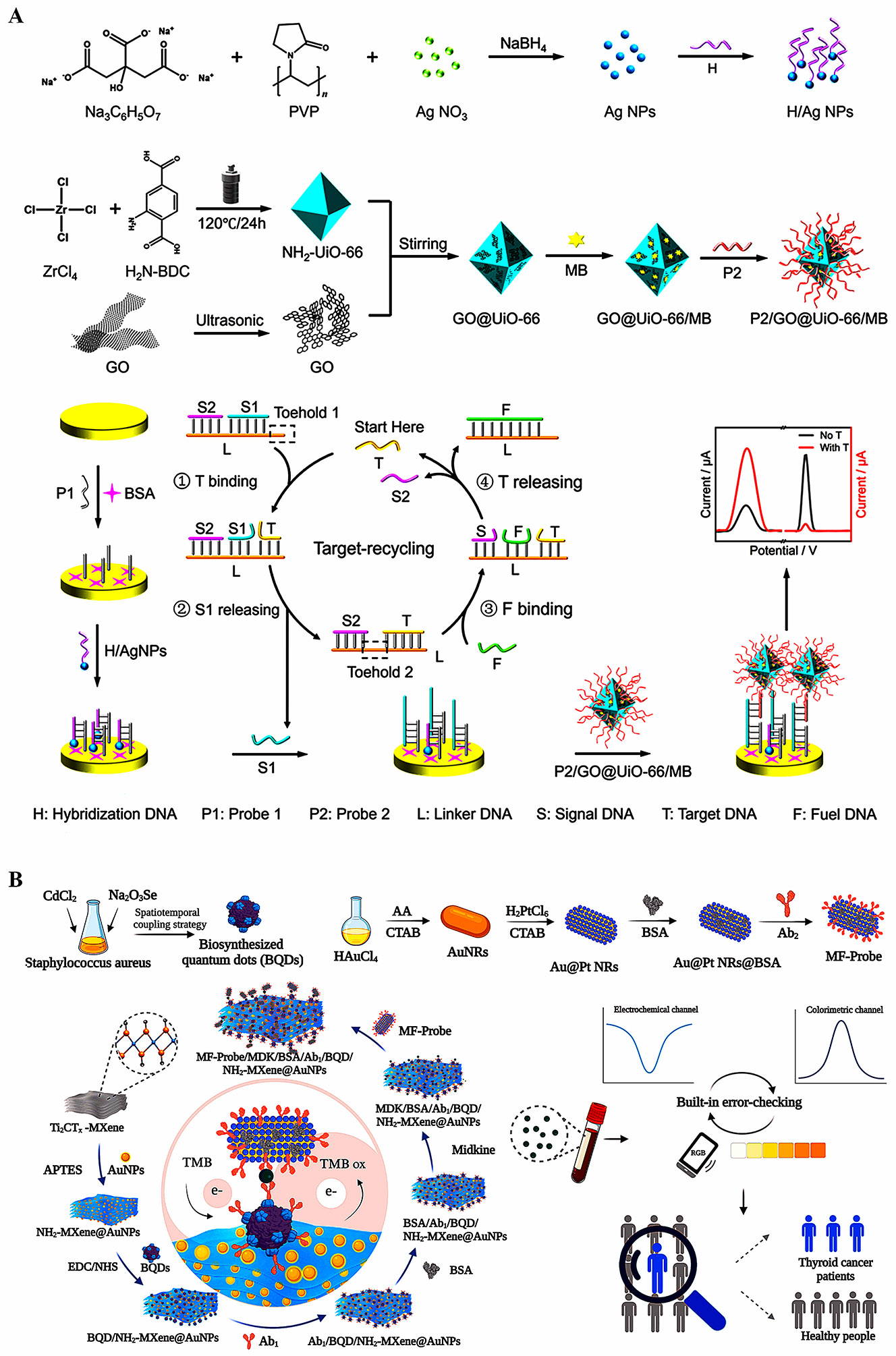
**A:** Architecture and signal transduction principle of the self-calibrating electrochemical biosensor for ORAOV1 detection based on fuel-driven DNA recycling and ratiometric output; Reuse License Number: 6190550650128; [85]; and **B:** Oriented self-assembly of the biosensing interface and its triple-mode error-checking detection mechanism for MDK quantification; Reuse License Number: 6190550908411; [107].

Oral squamous cell carcinoma (OSCC) is the most common and one of the most aggressive oral malignancies, with more than 300,000 new cases diagnosed annually worldwide, and early diagnosis remains a major clinical challenge [82,83]. Oral cancer overexpressed 1 (ORAOV1) is a promising oncogenic biomarker that is significantly upregulated in OSCC and detectable in noninvasive specimens such as saliva, highlighting its potential utility for early screening and diagnosis [84]. To aid the diagnosis of OSCC, Li and colleagues developed an amplified, self-calibrated electrochemical biosensor for the detection of ORAOV1 by combining nanoscale engineering and DNA molecular machines, with ratiometric electrochemical approaches [85]. The sensor interface employed spherical silver NPs (AgNPs, ~6.8 nm) surface-functionalized with DNA, together with a graphene oxide coated UiO-66 metal–organic framework nanocomposite (GO and UiO-66, ~100 nm, octahedral), which interacted with methylene blue (MB) (Fig. 3A). The AgNPs functioned as robust electroactive tags through the Ag/Ag^+^ redox couple, while the GO@UiO-66/MB composite served as a signal amplifier due to its high MB loading capacity and electrical conductivity, as well as a ratiometric counterpart to the AgNPs signal. The biosensor used a target-triggered, fuel-based DNA molecular machine that supported continuous recycling of the target sequence, ORAOV1, thereby amplifying the signal without enzymes. By incorporating self-calibration, the system minimized false negatives and false positives arising from nonspecific adsorption, probe variability, or instrumental drift. This self-calibration was achieved via a ratiometric electrochemical analysis, in which the ratio of the dual signals produced from AgNPs (Ag/Ag^+^) and from GO@UiO-66 functionalized with MB compensates for variations due to nonspecific binding and probe concentration changes. SWV was the primary method used for signal transduction. The biosensor possessed a detection range from 0.01 fM to 1 pM, with a LOD of 0.28 aM. The sensor demonstrated high specificity, with negligible responses to mismatched and noncomplementary sequences. Real-sample analysis using human saliva samples yielded acceptable recoveries and low matrix interference.

### Platforms developed for the diagnosis of lung cancer

Lung cancer remains the leading cause of cancer-related mortality worldwide, largely due to its late-stage diagnosis and aggressive disease progression. According to the American Cancer Society, lung cancer accounts for a substantial proportion of cancer deaths in both men and women, exceeding the combined mortality of several other major cancers. Early detection is critically important because survival rates are significantly higher when tumors are identified at localized stages than in advanced metastatic disease. Recent advances in screening strategies, particularly low-dose computed tomography (LDCT), along with emerging molecular and biomarker-based diagnostic approaches, have shown promise for improving early diagnosis and reducing lung cancer mortality. Therefore, enhancing early-detection technologies and improving screening accessibility remains a key priority in global lung cancer control [86,87].

Non-small cell lung cancer (NSCLC) accounts for 80–85% of lung cancer cases and remains one of the leading causes of cancer-related death globally [88]. CTCs, which detach from primary or metastatic tumor sites into the bloodstream, are considered a promising biomarker for non-invasive cancer diagnosis and monitoring [89]. In a study conducted by Lui et al., a simple liquid biopsy platform was developed using nitrogen-doped carbon quantum dots (N-CQDs) and AuNPs (9–12 nm), which were modified with dual aptamers to electrochemically detect NSCLC CTCs, particularly H1299 and A549 cells [90]. The N-CQDs provided abundant carboxyl groups for aptamer conjugation, with high biocompatibility, whereas AuNPs served as electroactive reporters with high conductivity and sufficient redox activity. Dual aptamer modification targeting EpCAM and vimentin increased the probe’s ability to capture heterogeneous CTC subpopulations, thereby overcoming the limitation of single-marker methods. Detection relied on the collaborative use of aptamers conjugated to N-CQDs (E/V-apt-N-CQDs) toward efficient capture of CTCs and programmed cell death 1 ligand 1 (PD-L1) hairpin aptamer-conjugated AuNPs (PD-L1-apt-AuNPs), aiming to provide improved electrochemical signals. After capturing CTCs from peripheral blood, PD-L1 levels were measured without additional reagents. The PD-L1 aptamer hairpin undergoes conformational changes upon binding to PD-L1, thereby revealing a biotin site that enables specific immobilization of AuNPs on a streptavidin-modified screen-printed carbon electrode (SPCE). SWV was then used to record the reduction peak of AuNPs around + 0.7 V, corresponding with PD-L1 concentration on CTCs. The platform could detect PD-L1 concentrations as low as 2 ng mL^−1^, with a quantitative range of up to 500 ng mL^−1^. The biosensor demonstrated outstanding analytical performance, achieving capture efficiency above 90% across a wide range of CTC concentrations (5–1000 cells mL^−1^) in phosphate-buffered saline (PBS), THP-1 cell suspensions, and whole blood.

Among the promising oncogenic biomarkers, abnormal DNA methylation of the short stature homeobox 2 (SHOX2) and Ras-association domain family 1 A (RASSF1A) genes has been strongly associated with NSCLC [91,92]. Lu *et al*., developed a SERS-assisted biosensor that was capable of detecting the SHOX2 and RASSF1A genes simultaneously with high specificity and sensitivity [93].

Raman spectroscopy is an optical technique that probes the vibrational modes of chemical bonds within molecules. It provides a unique spectral fingerprint of compounds; however, it is an inherently weak process because the vibrational transitions probed are technically forbidden in the electric-dipole approximation [94]. SERS is a method that enhances Raman signal strength by leveraging the unique optical properties of resonant noble-metal nanoparticles. Raman signal scales approximately with E4, where E is the local electric field [95]. Resonant metallic nanoparticles, particularly those made of silver and gold, can support surface plasmons when excited with the appropriate laser wavelength and generate local field enhancements of up to three orders of magnitude at sharp tips or in interparticle gaps. Therefore, molecules bound to the surface of these plasmonic nanoparticles and subjected to enhanced near-fields can experience Raman signal enhancement factors of up to 1012 [96]. A second complementary process, called chemical enhancement, can also boost the Raman signal by up to two orders of magnitude when charge transfer occurs between the metal surface and the molecule, altering the molecule's polarizability and increasing the Raman cross section [97]. Nanoparticles engineered to generate intense localized electromagnetic fields under near-infrared excitation (e.g., 785 nm), a wavelength range with enhanced biological tissue penetration [98], can amplify Raman scattering sufficiently to retrieve spectral signals buried in up to 14 cm of biological tissue [99]. When functionalized with reporters that exhibit strong SERS cross-sections and distinct non-endogenous spectral features, plasmonic nanoparticles have been demonstrated as highly effective contrast agents for cancer imaging, owing to their low cytotoxicity and their accumulation at tumor sites via the enhanced permeability and retention (EPR) and/or active transport and retention mechanisms after intravenous injection. The following section explores recent developments in SERS tumor imaging and its combination with complementary imaging and sensing methodologies.

Lu *et al*.’s sensing platform utilized spherical nanocomposites with an average size of ~50 nm, comprising Fe_3_O_4_ decorated with Au NPs as the magnet separation material and two different SERS nanotags, Au surface modified with mercaptobenzoic acid (MBA) then capped with Ag and Au modified with trifluoromethylbenzoic acid (TFMBA) capped with Ag, as the SERS reporters. These components were functionalized with DNA probes and antibodies, respectively, enabling selective recognition of the target genes. The Au nanoparticle-coated Fe_3_O_4_ particles provided strong magnetic capture of DNA, enabling efficient target enrichment, while the MBA- and TFMBA-containing nanotags served as SERS reporters, each exhibiting distinct Raman signatures corresponding to a specific target gene. The detection process relied on a sandwich hybridization protocol, where the formalin-fixed paraffin-embedded (FFPE)-released DNA was introduced into the nanotag assay. Gene sequences of RASSF1A and SHOX2 hybridized with complementary DNA probes immobilized on Au nanoparticle-coated Fe_3_O_4_ particles, while secondary probes conjugated to the MBA- and TFMBA-modified nanotags bound to other regions of the same target DNA. This dual-probe design enabled the formation of DNA–nanotag assemblies, generating distinct SERS signals, MBA for RASSF1A and TFMBA for SHOX2. A standard glass flask containing PBS buffer was used as the reaction setup, and multiplex detection was performed with a Raman spectrometer. The sensor showed outstanding analytical performance, with a detection range from 10 pM to 100 nM, and LODs of 0.52 pM and 0.66 pM for RASSF1A and SHOX2, respectively. Selectivity studies confirmed that the sensor could reliably discriminate among fully complementary, mismatched, and noncomplementary DNA sequences, ensuring accurate detection. Reproducibility tests yielded a RSD below 5%, while stability studies demonstrated robust retention of Raman signals. Analysis of real samples extracted from FFPE tissues further demonstrated the platform’s clinical relevance and translational potential.

The EGFR L858R mutation is a significant biomarker, present in up to 40% of EGFR-mutant NSCLC cases, and serves as a key predictive marker, guiding the use of EGFR-targeted tyrosine kinase inhibitors [100]. Precise and ultrasensitive detection of mutations in ctDNA is vital for early diagnosis, prognosis, and monitoring therapy [101]. Recently, a bimodal biosensor was developed that combined dual CRISPR-Cas12a cascade amplification with fluorescence and electrochemical transduction for the detection of ctDNA [102]. The platform was equipped with streptavidin-coated magnetic beads (SMBs), which functioned as the solid support and were modified with biotinylated DNA probes. A hybridization chain reaction-based DNA tetrahedron (Td-HCR) was employed to improve hybridization kinetics and promote the formation of branched DNA nanostructures that served as amplifiers. The platform utilized two successive CRISPR-Cas12a activations. At the initial level, digestion-defying ctDNA carrying the EGFR L858R mutation activated Cas12a/CrRNA1, which cleaved biotin-labeled DNA probes and inhibited the immobilization of FAM-labeled Td-HCR products on SMBs. This resulted in low fluorescence intensity, creating a "signal-off" response. At the second level, the non-immobilization of Td-HCR products on SMBs prevented activation of Cas12a/CrRNA2 and thus no cleavage of T2 probes on the gold-modified GCE. This allowed hybridization with T3-conjugated CuCo-oxide nanosheets modified with AuNPs (CuCo-ONSs@AuNPs), resulting in a robust electrochemical signal, a signal-on response. Overall, the bimodal outputs provided reciprocal validation and suppressed false positives. The sensor exhibited wide dynamic ranges of 1 pM-1 μM for fluorescence, with a LOD of 14.36 fM, and 10 fM-1 nM for electrochemical measurements, achieving an exceptional LOD of 372 aM. High selectivity was demonstrated by negligible responses to other cancer-associated mutations, including PIK3CA, EGFR T790M, and exon 19 deletions, confirming specificity toward L858R.

### Platforms developed for the diagnosis of other cancers

Telomerase is a promising biomarker, as it is overexpressed in approximately 85% of human cancers, whereas in normal somatic tissues, its expression is tightly regulated or absent, with activity largely restricted to germline and select stem or progenitor cell populations, making it highly suitable for early cancer diagnosis [103]. Urine biopsy offers a particularly attractive, noninvasive, and easily accessible platform for early cancer detection and longitudinal monitoring; however, conventional urine biopsy approaches have been limited in diagnosing cancers originating from distant sites because endogenously generated biomarkers are unstable and rapidly cleared. Therefore, researchers have developed synthetic probes that can translate tumor-related information into credible urinary indicators [104,105]. In a recent study, a metabolically stable biomarker nanoprobe with an artificial nature was designed, composed of 20 nm-diameter spherical AuNPs and functionalized with chimeric nucleic acids that linked DNA and peptide nucleic acid (PNA) for *in vivo* biosensing [103]. While the DNA moiety contained both the binding strand and the telomerase primer (TP) strand, metabolic stability was provided by the PNA reporter strand. When administered to mice bearing MDA-MB-231 xenograft tumors, spherical AuNPs provided a large surface area for nucleic acid loading, enhanced tumor accumulation via the enhanced permeability and retention effect, and served as carriers for the functional probe. At tumor sites, active telomerase elongated the TP strand, inducing the release of PNA reporters into the bloodstream and subsequently into urine due to their small size (4 kDa, below the filtration threshold) and neutral charge. The detection mechanism relied on electrochemical DPV in a urine biopsy format. The gold electrode (GE) was treated with scaffold DNA and MB-labeled signal DNA as the sensing interface. Upon the addition of PNA reporters from urine samples, the PNA reporters competitively hybridized on the scaffold DNA for the displacement of the MB-labeled signal DNA due to the superior hybridization affinity of the PNA. The displacement led to a reduction in the methylene blue (MB) electrochemical signal, with the extent of signal decrease directly correlating with the concentration of PNA. EIS, gel electrophoresis, and MALDI-TOF mass spectrometry (MS) verified the efficient displacement mechanism and the robust recovery of the PNA in urine. Compared to traditional DNA-based probes, the AuNPs-based nanoprobe exhibited high loading efficiency, optimal hybridization conditions, and nuclease stability. Using triple-negative breast cancer xenograft mouse models, the system enabled tumor diagnosis via urine biopsy as early as six days after transplantation, substantially earlier than detection achieved with conventional ELISA assays. The DPV-based sensor achieved quantitative urine-based detection of PNA reporters at approximately 176 nM at 3 h after injection. The electrochemical signal exhibited a tight correlation with tumor volume, validating the efficacy of this approach for monitoring disease progression.

Midkine (MDK) is a neurotrophic, heparin-binding growth factor that plays a key role in tumor growth, metastasis, and drug resistance [106]. It is overexpressed in a wide range of malignancies, including melanoma, breast, lung, ovarian, and prostate cancers. Owing to its secreted nature and elevated circulating levels in cancer patients, MDK has emerged as a clinically relevant serum biomarker associated with tumor presence, progression, and recurrence. A triple-mode biosensor was developed using BQD/NH_2_-MXene@AuNPs nanohybrids and Au@Pt nanorod probes [107]. It was fabricated by anchoring 10 nm spherical AuNPs onto aminated MXene sheets, which provide high electrical conductivity and an expanded electroactive surface area. The biosynthesized quantum dots (BQDs) were covalently immobilized onto the NH_2_-MXene-modified AuNP surfaces through amide bond formation. Protein A present on the BQDs surfaces enabled oriented immobilization of the primary antibodies (Ab1) via Fc-Protein A interactions, ensuring optimal exposure of the antigen-binding sites. For signal amplification, a multifunctional probe (MF-probe) consisting of platinum-coated gold nanorods (Au@Pt nanorods) conjugated with secondary antibodies (Ab2) was employed (Fig. 3B). This probe provided enhanced electrochemical conductivity and peroxidase-like activity, leading to improved sensitivity and error-checking through triple-mode detection. The detection relied on a sandwich-type immunoassay, in which serum MDK selectively bound to immobilized Ab1 on the electrode and was subsequently recognized by the MF-probe. Formation of the insulating protein layer increased charge-transfer resistance and decreased the DPV signal. Additionally, the electrochemical response was further improved by the intrinsic catalytic activity of Au@Pt nanorods in the reduction of H_2_O_2_. EIS and DPV were used to validate the stepwise modification of the electrode. Additional confirmation was achieved through colorimetric detection using the TMB/H_2_O_2_ system and a smartphone-based readout, enabling built-in error checking by cross-comparing signals across multiple detection modes. The analytical performance showed a broad linear detection range from 5 fg mL^−1^ to 100 ng mL^−1^, with a LOD of 1.620 fg mL^−1^. The selectivity performance was evaluated in the presence of interfering proteins, including CEA, α-fetoprotein (AFP), prostate-specific antigen (PSA), and HER2, which had negligible effects.

**Fig. 4.**
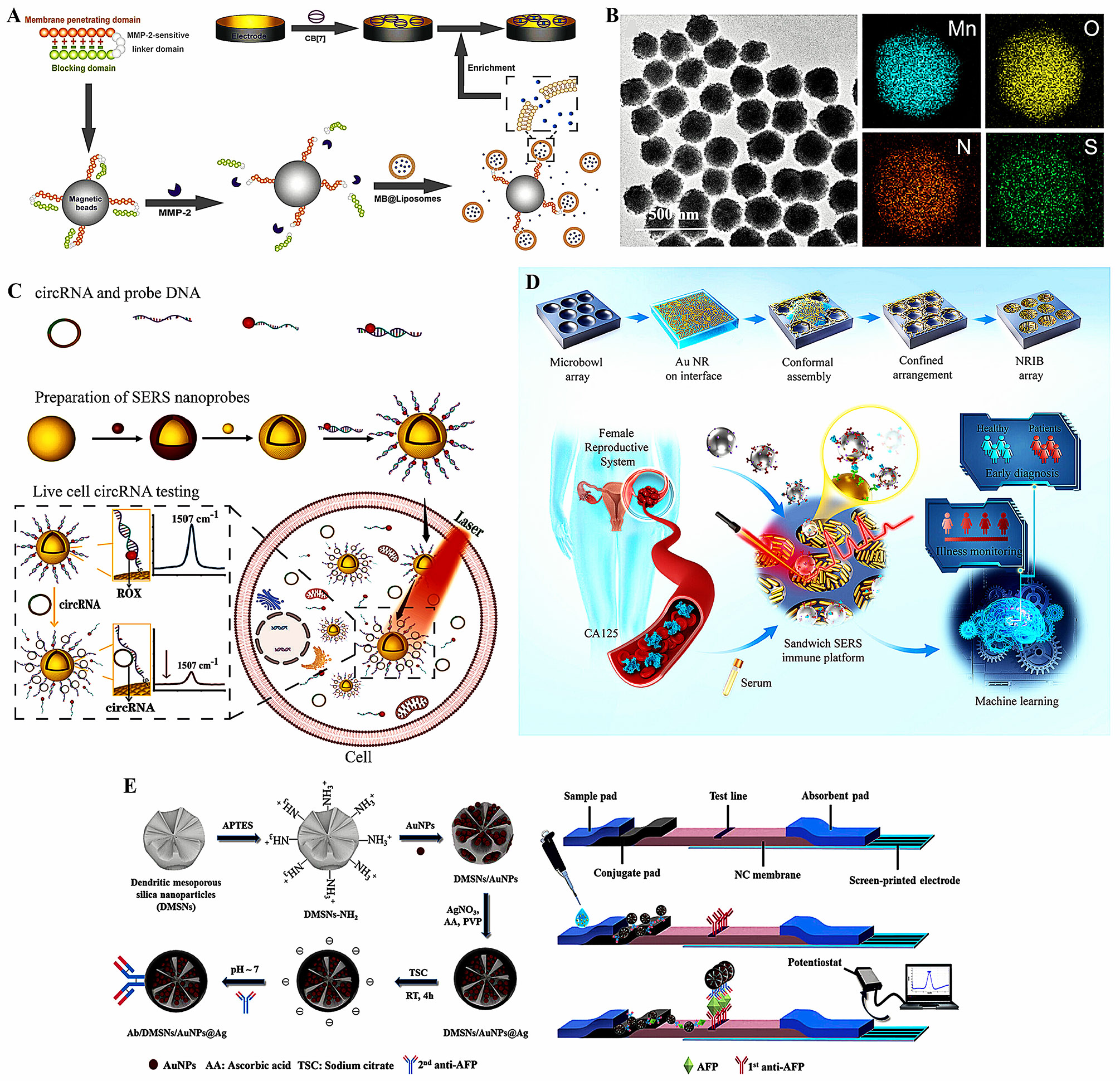
**A:** conceptual framework of the membrane-penetration-triggered electrochemical biosensor for MMP-2 detection; Reuse License Number: 6190551429081; [112]; **B:** morphological and structural characterization of chiral MnO_2_ supraparticles designed for MMP-9-responsive bioimaging; Reuse License Number: 6190541506326; [117]; **C:** design and intracellular sensing mechanism of the dual-signal SERS nanoprobe for quantitative circRNA detection in living cells; Reuse License Number: 6190560798996; [128]; **D:** design concept of the NRIB-based SERS immunoassay platform and machine learning-assisted quantification of CA-125; Reuse License Number: 6190570403259; [136]; **E:** configuration and working principle of the signal-boosted e-LFIA for AFP detection; Reuse License Number: 6190570077626; [81].

Matrix metalloproteinase-2 (MMP-2) is a zinc-dependent endopeptidase that degrades the extracellular matrix, thereby supporting tumor invasion, growth, and metastasis [108,109]. Elevated MMP-2 expression has been reported across a wide range of solid tumors, highlighting its potential utility as a broad cancer-associated biomarker, particularly for assessing tumor progression and invasive potential. [110,111]. Recently, an electrochemical biosensing method was developed using a tunable peptide probe, combined with signal amplification by liposome disruption, to enable highly specific detection of MMP-2 in cellular and clinical samples [112]. The sensor preparation involved spherical magnetic beads (~122 nm in diameter) surface-functionalized with a specially designed peptide probe (P1). This probe included a positively charged membrane-penetrating region, a negatively charged blocking segment, and an MMP-2-cleavable linker sequence (Fig. 4a). In the absence of MMP-2, electrostatic interactions between the domains kept the probe in the inactive conformation, preventing disruption of liposomes. When MMP-2 cleaved the linker, the membrane-penetrating domain was released, enabling interaction with MB-loaded liposomes (MB@liposomes). This cleavage induced membrane destabilization, causing the release of the MB molecule, which was then trapped on a cucurbituril[7]-functionalized electrode, producing amplified electrochemical signals detectable by SWV. Using MB@liposomes as electroactive signal reservoirs provided significant amplification, while magnetic bead separation ensured assay specificity and minimized interference from complex biological matrices. Transmission electron microscopy (TEM) and dynamic light scattering (DLS) confirmed the spherical nature and average size of the MB@liposomes (~143 nm after loading). EIS and cyclic voltammetry confirmed the functionalization of cucurbituril [7] electrodes and the efficient capture of MB molecules. Optimization experiments found the optimal loading of MB (10 μM), cleavage time (60 min), and interaction time between peptide-functionalized beads and liposomes (120 min). The biosensor exhibited a broad linear detection range from 50 fg mL^−1^ to 10 ng mL^−1^, with a LOD of 13.3 fg mL^−1^. Selectivity studies showed no significant interference from other cancer-associated proteins, including PD-L1, AFP, and CEA, while inhibition experiments using Ilomastat further confirmed the cleavage-dependent sensing mechanism.

Matrix metalloproteinase-9 (MMP-9) is a key extracellular protease involved in cancer growth, invasion, and metastasis, with high levels found in breast, colon, lung, and gastric cancers [113,114]. Thus, owing to its strong association with tumor progression and aggressive disease phenotypes, MMP-9 has attracted considerable interest as a cancer-associated biomarker, particularly for disease monitoring and prognostic assessment [115,116]. With this in mind, a renally-clearable MnO_2_ supraparticles (SPs) self-assembled with the MMP-9-cleavable peptide TGGGPLGVARGKGGC was fabricated, offering a dual-mode biosensing system for the detection of MMP-9 *in vivo* via circular dichroism (CD) and MRI [117].

CD spectroscopy is an ultrasensitive chiroptical technique that measures the differential absorption of left- and right-circularly polarized light by optically active biomolecules, enabling detailed characterization of conformational structures in proteins, nucleic acids, and other chiral biomacromolecules [118]. In cancer diagnostics, CD has emerged as a valuable analytical tool for detecting disease-associated structural alterations in biomolecules, such as conformational changes in circulating DNA, miRNAs, and tumor-related proteins, thereby providing insight into molecular signatures of tumor progression and facilitating the development of label-free biosensing strategies for early cancer detection [119,120].

MRI is a non-ionizing imaging technique that exploits the nuclear spin of hydrogen nuclei in water and lipids within biological tissues [121,122]. When proton spins are excited by radiofrequency pulses in a strong magnetic field, they are perturbed from equilibrium and subsequently relax back to alignment with the magnetic field through longitudinal (T1) and transverse (T2) relaxation processes. These relaxation properties, together with proton density, determine tissue contrast and enable differentiation between soft tissues [122]. MRI is widely used in oncology for tumor imaging; however, intrinsic contrast is often insufficient for early disease detection or molecular-level characterization [123]. Nanoparticle-based contrast agents have therefore emerged as powerful tools to enhance MRI sensitivity. Superparamagnetic nanoparticles primarily enhance MRI contrast by shortening T2/T2* relaxation times, while nanoparticles that release paramagnetic ions (e.g., Gd^3+^ or Mn^2+^) produce positive contrast through T1 relaxation shortening [124]. In addition to improving anatomical contrast, nanoparticles can also enable molecular imaging through surface functionalization with targeting ligands such as antibodies and peptides [125,126].

54 For MMP-9 detection, MnO_2_ SPs (approximately150 nm in diameter), were assembled from ultra-small (2-3 nm) MnO_2_ NPs linked via peptide dimers. The design incorporated chirality through the peptide ligands, with L-enantiomers showing improved tumor targeting over D-enantiomers due to their higher affinity for CD47 receptors overexpressed on tumor cells (Fig. 4b). Following intravenous administration, the SPs accumulated at tumor sites through iRGD-mediated targeting, where MMP-9 enzymatically cleaved the peptide linkers. This cleavage triggered the disassembly of the SPs into ultrasmall MnO_2_ NPs (<5 nm), which retained strong chiroptical activity and were efficiently cleared through the urinary system. The cleavage not only enabled quantitative *in vivo* monitoring of MMP-9 levels through urine but also provided high-resolution imaging of the tumor sites. Detection was dependent on the dual readout of the CD and MRI signals. CD enabled precise, sensitive detection of a change in chirality during the breakup of the SPs, while T1-weighted MRI provided improved visualization of MnO_2_ nanoparticle accumulation. Both approaches showed linear output signals versus MMP-9 concentrations between 0.01 and 10 ng mL^−1^, with LODs of 0.0054 ng mL^−1^ and 0.0062 ng mL^−1^ for CD and MRI, respectively. *In vitro* experiments showed high specificity for MMP-9 over other proteases, such as thrombin, MMP-1, MMP-2, and MMP-8, as no other protease disassembled the NPs except MMP-9. *In vivo* experiments on mouse models of breast cancer and colon cancer showed specific tumor accumulation in the time interval between 1.5 h after the injection and the general clearance after 48 h, as verified by imaging (CD and MRI) and urinary assay. This dual-mode nanosensor demonstrated high stability and reproducibility, with CD and MRI outputs showing robust correlations across multiple measurements, and no significant genotoxic effects on the animals were observed. The chiral SP-based, cleavage-mediated modular design provided high selectivity and sensitivity while enabling noninvasive cancer diagnosis through urine-based approaches. By integrating MRI with chirality-induced CD signals, the platform enables robust early detection and monitoring of MMP-9, advancing clinical screening and MMP-9-guided cancer treatment monitoring toward practical application.

Circular RNAs (circRNAs) are a recently recognized class of noncoding RNAs that have attracted significant interest in cancer biology due to their high stability, relative abundance, and capacity to regulate gene expression [127]. Their dysregulation has been strongly linked to tumor initiation, cancer progression, and metastasis; therefore, circRNAs have significant potential as biomarkers for cancer diagnosis and monitoring. A SERS-based assay was developed for circRNA detection using the plasmonic properties of a gold core–shell nanocomposite (~43 nm) functionalized with DNA probes [128]. The gold core–shell nanostructure was labeled with 4-mercaptobenzonitrile (4MBN), which exhibits a distinctive spectral signature that was strongly amplified within the intershell gap between the inner and middle Au layers. In addition to enhancing Raman signals through strong electromagnetic field coupling, the core-shell design also improved spectral stability and reproducibility. DNA probes were covalently immobilized on the outer Au surface, enabling specific hybridization with the target circRNA sequences (Fig. 4c). The detection principle relied on hybridization-induced modulation of the SERS signal. Hybridization of complementary circRNA targets with the DNA-functionalized nanocomposites brought the targets into close proximity to the 4MBN Raman reporter, enhancing the SERS signal and enabling quantitative detection. Proof-of-concept studies demonstrated the sensor’s strong analytical performance, achieving a wide linear detection range from 1 pM to 100 μM with an LOD of approximately 0.043 pM. Reproducibility tests showed consistent SERS signals across multiple measurements, while stability studies confirmed the structural integrity of the nanocomposites throughout the experiments. Selectivity evaluations further demonstrated the sensor’s ability to clearly discriminate complementary circRNA sequences from mismatched and noncomplementary sequences.

Carcinoembryonic antigen (CEA) is one of the most clinically established tumor markers, with elevated serum levels frequently observed in colorectal, gastric, esophageal, and lung cancers. [129,130]. As a soluble glycoprotein in the bloodstream, CEA is a significant biomarker for early diagnosis, prognosis, and monitoring therapeutic response. Traditional methods such as ELISA often suffer from limited sensitivity and low reproducibility, especially for early-stage detection [131]. To circumvent this limitation, an electrochemical immunosensor was developed that integrated the advantages of a hydrogen-bonded cobalt-porphyrin framework (Co-HOF) with antibody-based recognition, achieving high signal amplification and exceptional specificity [132]. The Co-HOF nanomaterial was synthesized as rod-shaped particles with an average size of approximately 1.17 μm, offering high surface accessibility, abundant active sites, and strong electrocatalytic activity. For the preparation of the sensing interface, a GCE was used, which was modified by electrodepositing AuNPs to provide high conductivity and facilitate stable attachment of capture aptamers via Au-S covalent bonds. Then, antibodies specific to CEA were attached onto the Co-HOF nanomaterial to develop a dual-recognition system that enhanced capture efficiency and suppressed nonspecific interactions. Detection was performed in a sandwich-type format, in which CEA antigens in human samples were initially captured by an aptamer attached to the electrode surface and subsequently recognized by the Co-HOF-antibody nanocomposite. This dual-capture strategy enhanced specificity while also enabling catalytic signal amplification, as the Co-HOF nanomaterial facilitated electron transfer and increased the electrochemical response current. DPV was used as the detection technique, in which the formation of the aptamer-antigen-antibody complex produced measurable changes in current that were proportional to the concentration of CEA. The immunosensor showed excellent analytical performance, with a wide linear range from 0.001 to 50 ng mL^−1^ and an ultralow LOD of 0.22 pg mL^−1^, demonstrating sensitivity well beyond that of commercial clinical assays. Selectivity experiments indicated no significant interference from other serum proteins, including AFP, PSA, and CA-125, thus verifying the platform’s specificity. Clinical validation using CRC patient serum samples validated the platform’s high diagnostic potential, with findings that agreed very well with the ELISA results.

CA-125 is a well-established serum biomarker in ovarian cancer and is routinely used for disease monitoring and prognostication; however, its diagnostic utility is limited by reduced sensitivity in early-stage disease and lack of specificity [133,134]. Commonly used CA-125 immunoassays have limitations, including a narrow linear range and susceptibility to the high-dose hook effect, particularly at high antigen concentrations, leading to false-negative results and diminished clinical reliability [135]. A recent study introduced an ultrasensitive SERS immunoassay platform based on nanorod-in-bowl (NRIB) arrays coupled with machine learning, capable of precise quantification of CA-125 across a wide dynamic range [136]. The sensor was prepared by embedding Au nanorods into periodically spaced SiO_2_ microbowls through a three-phase interfacial assembly process, then using a PDMS-assisted swabbing method to improve uniformity and hotspot distribution density (Fig. 4d). In this manner, the 3D plasmonic NRIB architecture generated abundant electromagnetic “hotspots” that enhanced light confinement, resulting in strong Raman signal amplification with an analytical enhancement factor of up to 1.09 × 10^8^. AgNPs were doped with 4-mercaptobenzoic acid (4-MBA) as the Raman reporter (Ag@4-MBA) and functionalized with anti-CA-125 antibodies to form SERS nanotags. Using these nanotags and antibody-functionalized NRIB substrates, a sandwich immunoassay was constructed in which antigen–antibody interactions localized the nanotags within the three-dimensional hotspot regions. This close proximity enhanced the 4-MBA SERS signal, with peak intensities directly proportional to CA-125 concentration. Detection was based on the observation of the Raman peaks of 4-MBA at 1075 cm^−1^ and 1586 cm^−1^, increasing proportionally with the level of CA-125 in serum samples. Significantly, the standard fitting was constrained by saturation of adsorption at the highest concentrations. Thus, convolutional neural network models were used to extract spectral features and provide accurate concentration estimates across the entire concentration range. This machine-learning-based analysis not only eliminated nonlinearity but also improved robustness to variability in the spectra. The biosensor exhibited an outstanding detection range from 1 to 5000 mL^−1^, with an LOD of 1 mL^−1^. Validation of the performance with clinical serum specimens from healthy volunteers and ovarian cancer patients demonstrated significant overlap with hospital test results. Testing demonstrated excellent reproducibility, with relative standard deviations below 10% across repeated measurements, and sufficient stability for up to 3 h following antigen-antibody interaction, which is critical for biological analyses. The system also showed high selectivity, as no notable SERS signal was found for interferent proteins such as CEA, AFP, and CA199.

Nanoparticle-enhanced laser desorption/ionization MS (NPELDI-MS) for metabolic fingerprinting is another research snapshot that has recently been considered. Cancer displays altered metabolic activity to sustain its growth; the resulting shift in the type and abundance of metabolites away from a healthy profile is a powerful diagnostic tool. The Warburg effect is a common example of such a metabolic shift, in which cancer cells metabolize glucose to lactate, despite reduced ATP yield, to produce metabolic intermediates as building blocks for the anabolic pathways needed for cell proliferation [137]. This change in metabolic activity is not a single biomarker for cancer diagnosis, but the resulting global change in metabolic profile could serve as a means of diagnosing cancer using multivariate analysis without requiring full molecular identification [138-140]. The most effective way to characterize the global small-molecule profile of a biological sample is MS, which measures the mass-to-charge ratio of ionized molecular fragments from the biological sample of interest [141]. The metabolic profile can then be used to classify samples as healthy or tumorigenic via multivariate analysis of the training data. Sample preparation is a key step before MS analysis: small metabolite molecules must be separated from larger macromolecules such as proteins, DNA, and carbohydrates, which can suppress ionization efficiency and reduce accuracy [141]. Resource-intensive specialist methods, such as liquid or gas chromatography, are typically used for sample preparation, requiring several hours per sample and large sample volumes for effective purification [142-144]. Recently, Wang et al. have shown that traditional small-molecule purification methods can be replaced with ferric oxide (Fe_3_O_4_) nanoparticle-based methods, which effectively separate metabolites from protein mixtures by size-exclusion [140]. NPELDI-MS then uses these ferric oxide NPs as a matrix to absorb laser energy and mediate ion formation in a rapid, low-sample-consumption method for metabolic fingerprinting from human serum samples. The ferric oxide NPs are synthesized using the solvothermal method, during which irregular Fe_3_O_4_ nanocrystals fuse to form larger particles with interparticle gaps. This results in NPs with rough nanoscale surfaces and 3.5 nm-sized pores, which, when mixed with a biological sample, act as sieves for larger protein molecules and trap small metabolites via surface adsorption. Elemental mapping of the Fe_3_O_4_ NPs after treatment with test solutions showed an 8-fold increase in metabolite signal intensity relative to protein signal intensity, confirming effective sieving and enrichment of the smaller metabolites by the NPs [138]. The LOD for NPELDI-MS was tested on a panel of small metabolites, and an impressive LOD of 0.07 μM was reported for aspartic acid, and more recently, this has been lowered further for metabolites such as alanine acid (11.2 pM) and glutamic acid (6.8 pM), confirming the method is suitable for accurate metabolic fingerprinting [138,140].

By applying machine learning to metabolic spectra, NPELDI-MS has been used to distinguish gastric cancer patient samples from benign samples, achieving an area under the curve (AUC) of 0.979 across 284 paired tissue samples [140]. Furthermore, the method effectively characterized tumors as HER2-positive or HER2-negative (AUC = 0.962), a key marker for treatment decisions, as HER2-positive tumors may respond to monoclonal antibody therapies such as trastuzumab. This metabolite separation method enabled faster HER2 subtyping in gastric cancer samples, whereas standard methods, such as immunohistochemistry and fluorescence in situ hybridization, are multiday processes. In contrast, the NPELDI-MS approach achieved total processing times of 40 min and was paired with accurate machine-learning data interpretation, thereby avoiding time-consuming expert-led data analysis [138]. NPELDI-MS has also been applied to serum metabolic fingerprinting in ovarian cancer [139]. In a larger-scale study including 662 ovarian cancer patients, 563 benign ovarian disease patients, and 207 healthy controls, an NPELDI-MS test requiring 150 nL of serum achieved an AUC of 0.87–0.89, effectively distinguishing malignant from benign ovarian growths. The NPELDI-MS method showed improved diagnostic performance compared to analyzing a single conventional ovarian cancer biomarker, such as cancer antigen 125 [139].

A promising aspect of the NPELDI-MS method is its applicability to diagnosing multiple cancer types in a single test. Wang et al. effectively diagnosed pancreatic cancer, gastric cancer, and colorectal cancer using the same experimental framework and methods [140]. They demonstrate consistent biomarker trends across the different cancers, identifying four key biomarkers: upregulation of glucose and glycerol, and downregulation of malic acid and galactitol. The broad applicability of mass spectrometry for screening across multiple cancer types in a non-targeted manner uniquely positions it as a promising diagnostic test, while the innovation of using Fe_3_O_4_ to separate and concentrate small metabolites could enable the technology to be applied more rapidly and in resource-poor regions.

Table 1 presents the most recent nanomaterial-based sensing platforms developed for cancer diagnostics.

## Recent innovative nanotechnologies for cancer imaging

This section examines several recent case studies that demonstrate how nanoparticles with improved targeting, tumor penetration, and clearance are being developed for *in vivo* SERS, radionuclide, photoacoustic, luminescent, and fluorescent cancer imaging.

### Intraoperative SERS imaging of tumors

**Fig. 5.**
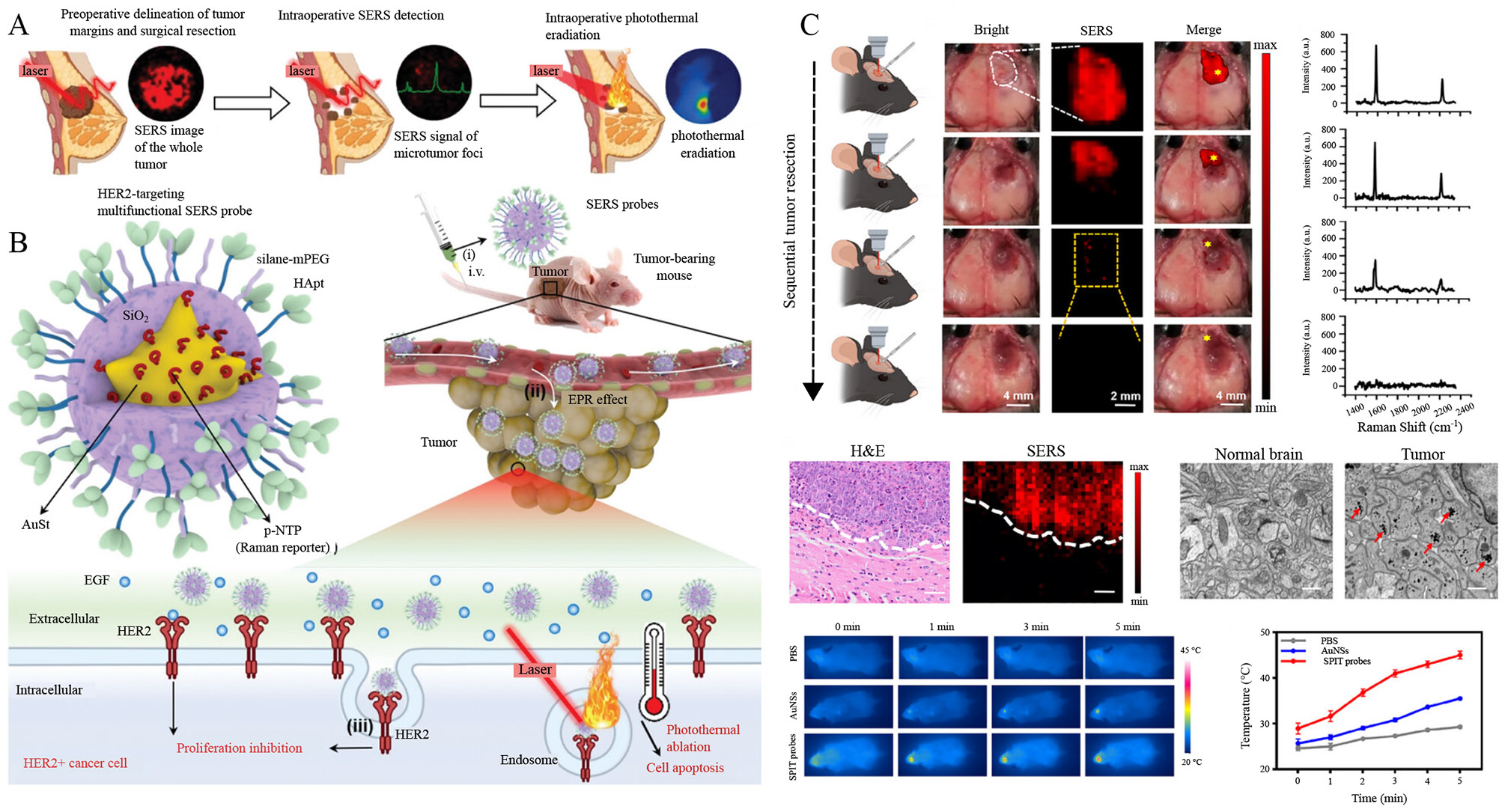
A: An overview of SERS-guided HER2+ breast-conserving surgery, the procedure starts with preoperative SERS tumor margin delineation, followed by intraoperative imaging and ablation of cancer foci post resection [189]. Printed with permission from Wen et al and John Wiley and Sons. B: Schematic of nanoparticle probe developed for SERS-guided HER2+ breast-conserving surgery comprised of a plasmonic gold nanostar core functionalized with Raman reporter molecule p-NTP and encapsulated by a silica shell protected with PEG molecules and conjugated to an aptamer specific to HER2. The nanoparticle probes accumulate in the tumor via the enhanced permeability and retention (EPR) effect and are then endocytosed by cancer cells upon binding to the HER2 membrane protein. Irradiation with an 808 nm laser selectively heats the nanoparticles via light absorption and induces tumor cell apoptosis [189]. Printed with permission from Wen et al. and John Wiley and Sons. C: “SPIT” probes applied to guiding glioblastoma resection *in vivo* and follow-up photothermal therapy on residual foci [194]; the top row illustrates SERS-guided brain surgery in a mouse model after SPIT probes accumulate at the tumor site. The distinct spectral fingerprint of the SPIT probe aids the surgeon in confirming the complete removal of all remaining tumor cells and foci from the resection area. The central row shows that SPIT probes are confined to the tumor tissue; the histological tumor boundary matches the Raman heat map, confirming that the signal only originates from tumor tissue. TEM images also clearly show SPIT probes in the intracellular space of tumor tissue, not in healthy tissue. Finally, the lowest row shows thermal camera images of mice during photothermal therapy: SPIT probes successfully heat tumors to +40 °C to induce apoptosis, while only modest temperature elevation is achieved in the control groups. Printed with permission from Xu et al. and John Wiley and Sons.

Breast cancer is the most commonly diagnosed malignancy among women worldwide, and although breast-conserving surgery offers clear survival and cosmetic advantages over mastectomy, incomplete excision of foci continues to drive local recurrence and poor long-term outcomes [187]. Intraoperative precise delineation of tumor margins and eradication of residual HER2-positive microfoci, could improve tumor removal while preserving healthy breast tissue [188]. Wen et al., [189] created a theranostic SERS nanostar probe that targets HER2, a surface protein overexpressed in certain cancers including breast cancer (Fig. 5A-B). The nanostar probes enabled precise intraoperative SERS mapping of tumor margins followed by photothermal ablation of residual microscopic foci in the resection bed. The synergistic properties of the gold nanostars (AuNStars) improved breast-conserving surgery outcomes in a HER2^+^ SKBR-3 mouse model. AuNStars resonant at ~760 nm were functionalized with p-nitrobenzenethiol and encapsulated in silica. Polyethylene glycol (PEG) was attached to the silica shells, which were then functionalized with HER2 aptamers at an average density of approximately 55 strands per nanoparticle, resulting in a final hydrodynamic diameter of ~254 nm. *In vitro*, nanoprobe uptake was shown to be higher in the HER2-positive cell line SKBR-3 than in the HER2-negative line MCF-7, demonstrating effective targeting. The probes demonstrated a photothermal efficiency of 49% under irradiation at 808 nm. *In vivo*, SKBR-3 xenograft-bearing mice were intravenously administered with 2.5 mg/kg of probe, and SERS mapping was utilized for pre- and intraoperative tumor margin delineation. Microfoci in the resection bed were then identified by SERS mapping, and localized photothermal heating with an 808 nm laser was used to ablate the foci. Aptamer HER2 targeting was shown to be effective *in vitro* and yielded a strong SERS signal *in vivo* to delineate tumor margins while also highlighting residual microfoci. Combining SERS-guided resection with mild photothermal ablation improved outcomes with no recurrence over 14 days and ~100% survival at 30 days. The authors report negligible *in vivo* toxicity in the short term and high nanoparticle stability. Limitations of the study include reticuloendothelial uptake of the probes and a lack of long-term studies to assess the health impact of residual probes.

Glioblastoma (GBM) is the most aggressive primary brain tumor and has poor patient outcomes due to its resistance to therapies and high recurrence [190]. The median survival of GBM patients is 12–15 months [191] and 80% of tumor recurrence is within 2 cm of the resection margins - indicative of missed malignant tissue during surgery [192]. NPs contrast agents for image-guided surgery are a promising technology for improving GBM resection by enhancing the removal of microfoci and increasing the rate of complete resection.

Lu and colleagues., created biomimetic near-infrared II (NIR-II) SERS tags by coating Au nanorod-Au/Ag nanoframes (a nanorod positioned within a gold cubic frame) with a combined red-blood-cell and U87-MG membrane, and the SERS dye IR-1061 [193]. These biomimetic tags targeted glioblastoma, enabling stepwise intraoperative resection guided by SERS in an orthotopic mouse model of glioblastoma, demonstrating complete tumor removal and improved surgical outcomes. Nanoframes were synthesized by the overgrowth of Ag and Au on Au nanorods, followed by selective oxidative etching, which created a hollow Au–Ag frame around a solid gold nanorod (AuNR) core, resulting in strong NIR-II plasmon bands. IR-1061 was used as the Raman reporter. The authors created hybrid membranes by fusing red blood cell and U87-MG membranes and verified fusion using a FRET fusion assay. The AuNR-Au/Ag nanoframes were coated in the hybrid membranes, and biocompatibility was confirmed in U87 cells and bEnd.3 cells. The ability of the SERS nanoframes to penetrate 3D spheroids was confirmed using SERS mapping before *in vivo* studies demonstrated NIR-II SERS could guide stepwise resection at 12 h post-injection. The nanoframes tunable plasmon band showed maximal NIR-II SERS enhancement when plasmon resonance was blue-shifted to 920 nm relative to excitation at 1064 nm. A SERS LOD of 5.87 μg mL^−1^ was measured, and signals were photostable over 60 min continuous illumination. The authors suggested that hybrid-membrane coating confers immune evasion and homotypic targeting, yielding high GBM tumor accumulation with tumor-to-normal tissue ratios up to 5.94 ± 1.71 and precise delineation of microtumors (~110 μm). *In vivo* NIR-II mapping achieved a penetration depth of ~5 mm and guided complete GBM resection, verified by histopathology. This study combines deep-tissue-penetrating, low--background NIR-II SERS excitation with biomimetic targeting to enhance surgical accuracy.

Building on the use of NIR-II SERS nanoprobes for precise intraoperative tumor visualization and complete resection, subsequent strategies have expanded SERS-guided platforms to not only delineate surgical margins but also actively address residual disease through integrated therapeutic modalities. Xu et al., present probes that combine SERS (S), photothermal therapy (P), and immunotherapy (IT) (“SPIT” probes) to improve glioblastoma treatment with intraoperative imaging and post-surgical therapy (Fig. 5C) [194]. SPIT probes are gold nanostars functionalized with a SERS reporter molecule and coated with macrophage membranes engineered to overexpress signaling regulatory protein α (SIRPα). CD47 is commonly overexpressed on tumor cells and binds SIRPα to inhibit phagocytosis. Therapeutic disruption of this pathway represents an emerging immune checkpoint strategy. Recent approaches have combined intraoperative SERS margin mapping with photothermal ablation and CD47-SIRPα blockade to address residual glioblastoma following surgical resection. AuNStars were encoded with 4-mercaptobenzonitrile (4-MBN), a Raman reporter exhibiting a strong vibrational band within the cell-silent spectral window and subsequently cloaked with membranes derived from lentivirally engineered RAW264.7 macrophages overexpressing high-affinity SIRPα. The membrane also contained integrin α4 and Mac-1, which have been shown to aid blood-brain-barrier (BBB) crossing. The authors demonstrated BBB translocation of the SPIT probes using an *in vitro* transwell model composed of bEnd.3 endothelial cells to mimic the BBB, co-cultured with GL261 glioblastoma cells to assess tumor uptake. SPIT probes were administered *in vivo* to mice bearing orthotopic GL261-Luc glioblastoma tumors, followed by SERS-guided surgical resection (S) using 785 nm excitation and subsequent 808 nm photothermoal therpay (P); immunotherapy (IT) responses were evaluated by flow cytometry. In comparison to AuNStars coated with unengineered macrophage cell membranes, SPIT probes increased BBB crossing and GBM accumulation and demonstrated strong SERS signal in the cell-silent region at 2217–2228 cm^−1^ to support intra-operative margin mapping, and elevated tumor-bed temperature to ~45 °C under 808 nm excitation. SERS mapping also revealed residual microfoci, enabling extended resection. Combined SERS-guided surgery+P + IT cut recurrence to ~12.5% and doubled median survival to more than 70 days. Photothermal therapy induced immunogenic cell death, enhancing immune response by releasing tumor-associated antigens and damage-associated molecular patterns. SIRPα enhanced macrophage phagocytosis of cancer cells, tumor microenvironment immunity showed higher numbers of dendritic cells relative to the controls, the authors ascribed this to increased dendritic cell recruitment following treatment with SPIT. The study shows SPIT can achieve precise tumor margin delineation, targeted photothermal therapy, and enhance the local immune response to remove residual tumor cells post-resection. Limitations include high SPIT uptake in the reticuloendothelial organs with no clear excretion route.

### Combined SERS and positron emission tomography (PET) for in vivo immunotherapy tracking

Immunotherapies harness the body’s immune system to recognize and eliminate malignant cells. However, only a minority of patients respond to immunotherapy, and a rapid assessment of efficacy is vital for determining treatment plans or switching to alternative treatments when immunotherapy is deemed ineffective or counterproductive [195]. *In vivo* imaging of recruited immune cell populations during immunotherapy offers a potential avenue for real-time evaluation of treatment response [196]. Imaging multiple immunomarkers *in vivo* would be a useful tool in evaluating a patient’s response to immunotherapy. Cutshaw and colleagues engineered multimodal gold nanostars (MGNs) that combine immunoPET’s whole-body sensitivity with Raman’s high-resolution multiplexing potential to longitudinally track CD8^+^ T cells and NKp46^+^ Natural Killer (NK) cells in the whole body and tumor microenvironment [197]. MGNs predicted therapeutic response in “hot” CT26 tumors and resistance in “cold” 4T1 tumors during treatment with an anti-PD-L1 + anti-CD47 combination immunotherapy, with *ex vivo* conventional assays confirming immune recruitment. The platform enables earlier and more effective detection of immunomarkers than single-modality probes. MGNs were fabricated by conjugating anti-CD8 and anti-NKp46 monoclonal antibodies (mAbs), a different Raman reporter for each immune cell type, and 1,4,7-triazacyclononane-1,4,7-triacetic acid (NOTA)-chelated ^64^Cu onto gold nanostars to yield two different Raman fingerprints and a PET isotope for quantitative temporal readout of total nanoparticle accumulation in organs. *In vivo*, mice received intraperitoneal administration of MGNs and were imaged at 6, 20, and 48 h. Blocked controls received excess free anti-CD8/anti-NKp46 injections to test the specificity of MGN targeting. Raman multiplexing showed peak sensitivity at 6 h following MGN administration, followed by decreasing signals consistent with dynamic targeting and clearance. Blocked controls significantly reduced Raman and PET signal at the tumor site, demonstrating target specificity. *Ex vivo* immune fluorescence, cytokine analysis with Luminex, and flow cytometry assays corroborated *in vivo* findings that immunotherapy increased intratumoral CD8^+^ and NKp46^+^ infiltration in responders and decreased PD-L1^+^ tumor cells. In the poor-responder 4T1 model, longitudinal Raman/PET signals and *ex vivo* markers indicated treatment resistance. PET gave quantitative, whole-body imaging and sensitivity, whereas SERS provided real-time, orthogonal spectral channels for simultaneous CD8/NK tracking at high spatial resolution at the tumor site. This dual-marker probe system enabled the prediction of a tumor’s response to immunotherapy by multiplexing beyond immunoPET alone and cross-validating with a conventional assay. However, the challenges associated with quantifying SERS signals at depth in tissues could limit their clinical utility.

### miRNA-responsive SERS imaging

**Fig. 6.**
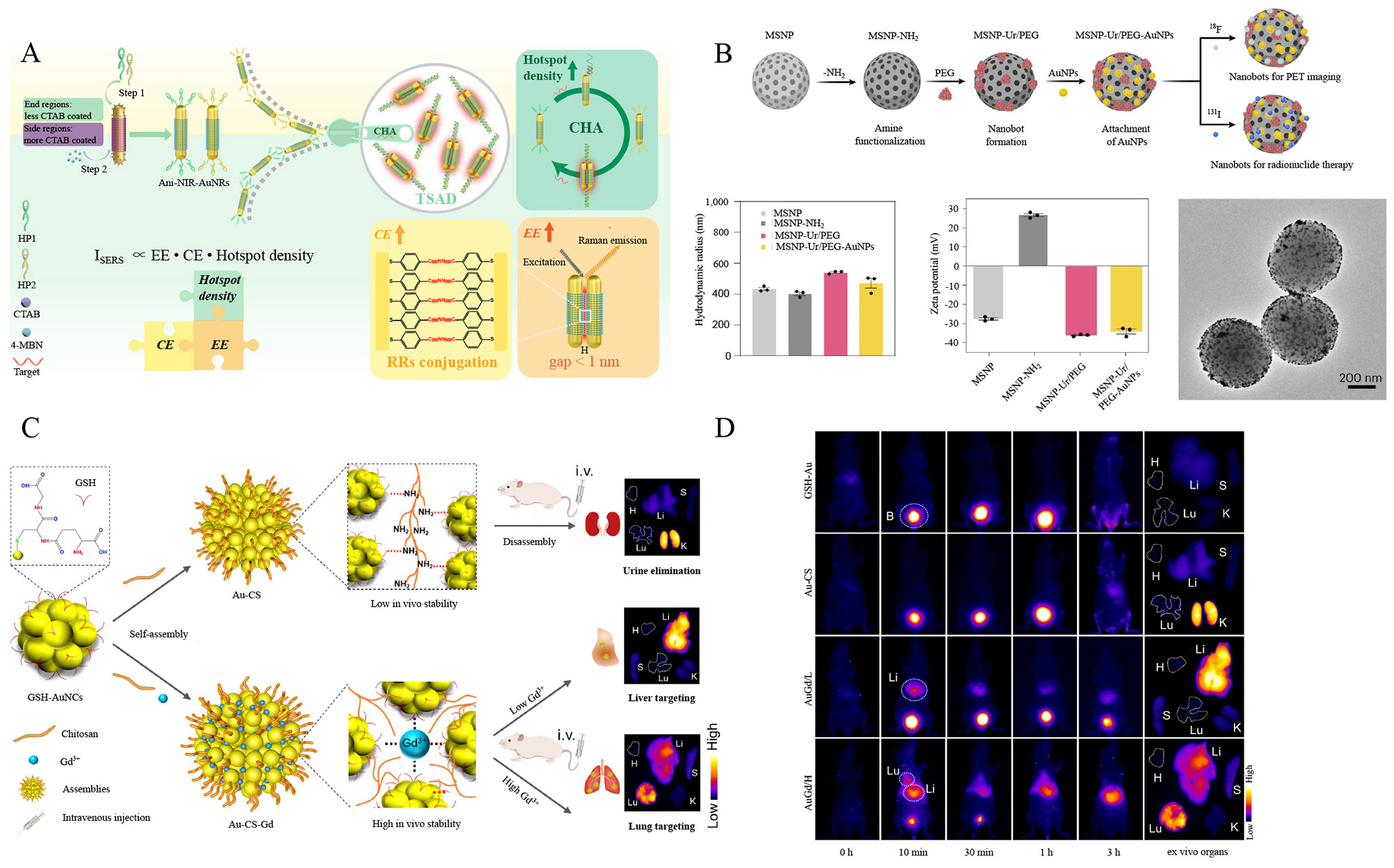
A: Schematic of miRNA-sensitive zippable AuNR probe fabrication and application to detect miRNA. Anisotropic functionalization was used to add DNA hairpins to the AuNR tips and Raman reporter 4-MBN to the AuNR sides. let-7d miRNA induced ‘zipping’ of AuNRs to create interparticle gaps containing 4-MBN that facilitated both chemical and electromagnetic SERS enhancement and sensitive miRNA detection *in vivo* [199]. Reproduced with permission from Lei et al. and American Chemical Society. B: The top row shows how mesoporous silica nanoparticles are functionalized with urease, followed by gold nanoparticles and an imaging or treatment radionucleotide. On the second row, DLS measurements show small increases in size as the nanoparticles are sequentially coated with urease and gold nanoparticles. The zeta potential of the nanoparticles fluctuates between positive and negative values with the addition of surface layers, resulting in a final value of −30 mV, which optimizes colloidal stability, circulation time, and biocompatibility. The bottom right panel in B shows a TEM image of the final, fully functionalized Nanobot [206] Reproduced from Simó et al., Nature Nanotechnology, 2024, licensed under Creative Commons Attribution 4.0 International License. C: Gold nanocluster fabrication for organ-targeted luminescent imaging. Synthesis conditions were optimized to target lung orthotopic tumors by tuning the Gd^3+^ concentration [216]; Chitosan nanocluster assemblies showed poor stability *in vivo*, leading to disassembly and excretion through the urine and resulted in a strong photoluminescent signal from the kidneys. However, when Gd^3+^ were incorporated, the assemblies were more stable *in vivo* and targeted the lung or liver, depending on the Gd^3+^ concentration. Reproduced with permission from Song et al. and American Chemical Society. D: NIR-II photoluminescence images of whole mice 0-3 h after intravenous injection of AuNCs, Au-CS, AuGd/L, and AuGd/H assemblies. The final column shows *ex vivo* NIR-II photoluminescence of major organs 3 h after injection. Reproduced with permission from Song et al. and American Chemical Society.

As highlighted previously, miRNAs regulate gene expression, and their dysregulation is associated with cancer [198]. miRNA is an attractive target for bio-responsive SERS NPs that ‘turn-on’ in response to interactions with dysregulated miRNAs [199]. One such approach is to functionalize the tips of anisotropic NIR-resonant Au nanorods with CHA-facilitating DNA hairpins, while functionalizing the longitudinal side of the nanorods with a nitrile Raman tag (Fig. 6A). Target miRNA “zippes” rods into uniform side-by-side dimers with ~0.88 nm gaps, creating intense, reproducible hotspots for bright, low-background NIR-SERS imaging in cells and live mice. The authors tested their sensor on let-7d, a miRNA downregulated in cervical cancer cells. AuNRs with an aspect ratio of 5 maximized NIR enhancement for dimers. CTAB-mediated anisotropic chemistry enabled the functionalization of rod tips with CHA hairpins. 4-MBN reporter molecules were selectively immobilized on the lateral surfaces of the nanorods via an optimized ligand exchange protocol, thereby avoiding the rod tips and preserving CHA functionality. Let-7d triggered end-to-end hairpin opening and toehold-mediated strand displacement, catalytically assembling many side-by-side dimers per target and boosting hotspot density. SERS peaks at 1561 and 2212 cm^−1^ are suitable for imaging, with the high-wavenumber Raman-silent region offering improved contrast and reduced biological background in cellular environments. *In vivo* imaging was performed using a confocal Raman microscope on a subcutaneous cervical cancer model following intratumoral injection of AuNRs. The sub-nanometer gaps formed following miRNA-triggered CHA strengthened near-fields at 785 nm, and 4-MBN chemical coupling increased chemical enhancement, strengthening the SERS signal after ‘zipping’. Let-7d was detected with LOD 0.15 fM and with a 1 fM-1 nM linear range, a significant improvement on FRET-based let-7d sensing. Both *in vitro* and *in vivo*, SERS signals were brighter, more spatially resolved, and longer-lived than fluorescence miRNA probes. *In vivo*, the SERS probes were responsive to both up- and downregulation of tumor-associated miRNAs. A key advantage of this approach is its on-off analyte sensitivity, a feature that is challenging to achieve with conventional SERS and enables quantitative bioimaging. However, the strategy relies on direct nucleic-acid access to the AuNR surface, which may be compromised by protein corona formation in biological environments. Moreover, the study employed intratumoral administration, and the feasibility of systemic delivery must still be established.

### Photoacoustic imaging of immunotherapy

Photoacoustic imaging is an optical and ultrasonic imaging technique that converts absorbed photons into acoustic waves through thermoelastic expansion [200,201]. Nanosecond-pulsed lasers are used to excite endogenous chromophores within tissue, such as hemoglobin, melanin, lipids, and water, which provide intrinsic photoacoustic contrast. Absorption of light by these molecules produces rapid transient heating, generating broadband ultrasonic waves that can be detected with ultrasound transducers and reconstructed into high-resolution images. This enables visualization of tissue structures, such as tumoral vasculature, and, when hemoglobin is targeted, allows assessment of tissue oxygenation [201]. Metallic nanoparticles, with their larger absorption cross-sections, absorb resonant light more effectively than endogenous chromophores, thereby generating clear contrast with background nanoparticle-free tissues [202]. When metallic nanoparticles are excited by resonant photons, the localized surface plasmon resonance, a collective electron oscillation, is initiated at the metal surface, and heat is conducted from the plasmonic particles to their surroundings on the picosecond timescale as oscillating electrons dephase and couple to phonons [203]. The efficient absorption and transfer of heat from the nanoparticles to the surrounding tissue maximizes the photoacoustic effect in tissues loaded with nanoparticles (i.e., tumor), thereby improving contrast. By optimizing nanoparticle size, shape, and dielectric surface coating, their plasmon resonances can be tuned to the near-infrared biological window (650–1350 nm), where photon penetration is greater due to reduced scattering [98]. Recently, these properties have made metallic plasmonic nanoparticles attractive agents for high-contrast deep-tissue photoacoustic cancer imaging and immunotherapy response monitoring.

Tracking T-cells is vital in cancer immunotherapies, helping assess therapy efficacy and whether immune cells are infiltrating the tumor. Maleimide-functionalization of silica-coated gold nanorods enables the NPs to covalently bond to and label T-cells. This approach could facilitate the tracking of T-cells during adoptive cell therapy using photoacoustic imaging [204]. In this study, the authors noninvasively tracked T-cell delivery and retention in tumors and simultaneously monitored blood biomarkers, enabling longitudinal assessment of treatment response *in vivo*. AuNRs (~8 ×50 nm) were synthesized with a plasmon peak at 1064 nm and coated with mesoporous-silica to enhance photostability for NIR-II photoacoustic imaging. By functionalizing the silica shell with maleimide groups, AuNRs were covalently linked to thiols on T-cell membranes. Gelatin phantoms were used to quantify photoacoustic performance, and the T-cell LOD per μl was evaluated. Primary murine OT-1 T cells labeled with AuNR were intravenously injected into C57BL/6 J mice bearing EG7-OVA (antigen-positive) or EL4 (antigen-negative) flank tumors. US and photoacoustic imaging was conducted over 7 days. US was used to measure tumor volumes and photoacoustic imaging at 1064 tracked T-cell accumulation. Multiwavelength photoacoustic sweeps with linear unmixing were used to measure oxygen saturation, oxygenated hemoglobin, and deoxygenated hemoglobin inside the tumor. The silica coating preserved the AuNR plasmonic properties and improved photostability, maintaining photoacoustic signal through up to 500 pulses at 20 mJ/cm^2^. Labeled T-cells generated a strong photoacoustic contrast, with statistically significant detection achieved at 125 cells/μL for cells incubated in OD = 10 solutions and 250 cells/μL for cells incubated in OD = 5. *In vivo*, antigen-positive mice exhibited a higher photoacoustic signal, indicating greater T-cell retention, reduced tumor volumes, a decrease in percentage oxygen saturation and oxygenated hemoglobin, accompanied by an increase in deoxygenated hemoglobin. AuNRs are exogenous absorbers that generate strong and spectrally distinct photoacoustic absorption at 1064 nm, which enables multiplexed functional readouts via spectral unmixing from endogenous absorbers such as hemoglobin. Advantages of the combined US/photoacoustic approach include nonionizing radiation, real-time measurements, and multiplex measurements with deep (NIR-II) sensitivity.

### Self-propelled NPs in imaging

A major barrier in nanoparticle imaging and therapy of solid tumors is nanoparticle penetration. Nanoparticles accumulate in tumors at sites of leaky vasculature via the EPR effect, but the dense tumor extracellular matrix, high interstitial fluid pressure, and heterogeneity can lead to uneven nanoparticle distribution, hindering imaging and therapy. MRI nanoparticle probes have recently shown improved tumor penetration by chemical modifications that enable self-propulsion via a Fenton reaction with hydrogen peroxide present in tumor microenvironments [205].

Sun and colleagues synthesized catalase-powered Janus catalytic NPs (FeO@mSiO_2_/Au-CAT) that self-propel in H_2_O_2_ concentrations found in hypoxic tumor microenvironments [124]. The NPs alleviate hypoxia, and their self-propulsion enables deeper penetration into solid tumors. Their absorbance of infrared radiation also facilitated photoacoustic imaging and photothermal therapy *in vivo*. Furthermore, NPs could be imaged by T2-MRI in a subcutaneous 4T1 tumor model, and combined photothermal (Au) and chemodynamic (FeO-Fenton) therapy inhibited the growth of subcutaneous 4T1 tumors in mice. Janus NPs (FeO@mSiO_2_/Au-CAT) were synthesized by coating FeO cores (~23 nm) with mesoporous SiO_2_ shells (125 nm), Au hemisphere deposition (~10 nm thickness), and catalase conjugation, creating NPs capable of self-propulsion and MRI contrast. Self-propulsion was demonstrated in 0–1 mM H_2_O_2_ and quantified by mean-squared displacement analysis. Photothermal properties at 808 nm were determined by UV–vis and thermal IR. Reactive oxygen species (ROS) production from Fenton activity was validated by electron spin resonance spectroscopy and methylene blue degradation. *In vivo*, 4T1-tumor bearing mice received intratumoral injections of NPs, and T2-MRI (r_2_) was used to track penetration. Photoacoustic imaging was used to assess hypoxia in the tumor following injection of NPs and the resulting Fenton reaction. FeO@mSiO_2_/Au-CAT showed strong NIR absorption and stable photothermal conversion. In the presence of H_2_O_2_, the NPs generated O_2_, decreased hypoxia-inducible factor 1α (HIF-1α) expression, and increased vascular saturated O_2_. FeO@mSiO_2_/Au-CAT had r_2_ ≈ 20.996 mM^−1^ s^−1^ and superior deep-tumor T2-MRI contrast compared with passive controls, indicating enhanced penetration. Intratumorally injected JNCRs irradiated with 808 nm significantly inhibited tumor growth. A dose of 2.5–10 mg kg^−1^ was determined to be biologically safe. The Au in the nanoparticle enabled photothermal therapy, FeO released Fe^2+^ in the acidic tumor microenvironment catalyzing ·OH production from H_2_O_2_ for chemodynamic therapy, and finally catalase-drivove self-propulsion of the nanoparticles improving intratumoral distribution. The study shows promise in tracking NPs *in vivo* using MIR, reducing hypoxia in the tumor microenvironment, and combining photothermal therapy with complementary therapies.

Self-propelled nanodevices can also exploit the unique microenvironment of specific organs to improve tumor penetration. Urease-powered self-propelled “nanobots’ have been shown to increase penetration and therapeutic efficiency in bladder cancer when intravesically delivered via a catheter. Simó *et al.* radiolabeled urease-powered mesoporous silica “nanobots” decorated with AuNPs, enabling autonomous motion in urea-rich environments of the bladder [206]. Intravesical administration of nanobots in an orthotopic mouse bladder cancer model demonstrated that the nanobots actively accumulate in, and penetrate, bladder tumors, enabling PET tracking and effective β-emitter (^131^I) radionuclide therapy, resulting in tumor reduction. Mesoporous silica NPs (~450 nm) were Stöber-synthesized, aminated, and glutaraldehyde-activated to conjugate urease and heterobifunctional PEG, and finally decorated with citrate AuNPs. For imaging, the particles were labeled with ^18^F; for therapy, ^131^I was conjugated to AuNPs (Fig. 6B). The nanobot motion was fueled by 300 mM urea. Orthotopic MB49 tumors were induced in C57BL/6 mice. Intravesical instillations of nanobots were incubated for 1 h before bladder emptying. PET imaging quantified *in vivo* accumulation and diffusion-weighted MRI provided tumor volumes. Scattered light-sheet microscopy of cleared whole bladders revealed the localization and quantification of nanobots and their depth penetration. Inductively coupled plasma mass spectrometry (ICP-MS) of Au validated PET-derived uptake. Therapeutic efficacy was tested at 1.85 and 18.5 MBq ^131^I-nanobots vs controls. Urease-powered nanobots produced an ~8-fold increase in tumor-site accumulation versus passive controls. Label-free scattered light-sheet microscopy exploited strong elastic scattering from AuNPs to map surface accumulation and measure penetration of nanobots into tumor versus healthy urothelium in 100 μm tissue sections. ^131^I-nanobots reduced tumor volume by ~90% with 18.5 MBq treatments and even 1.85 MBq arrested growth when fueled with urea, with no notable weight loss in animal subjects. Advantages of this appraoch include using microenvironment-specific fuel for self-propulsion in urine to reduce sedimentation and increase penetration depths and homogeneous distribution in tumors. Limitations include partial radiolabel detachment in urine and reliance on bladder urea, which makes it non-transferable to other tissues. The translation of this method to human intravesical therapy is yet to be demonstrated.

### Photoluminescent gold nanoclusters for tumor imaging

Photoluminescence can be used for cancer imaging with endogenous molecules or exogenous probes. Tumor metabolic activity upregulates naturally occurring fluorophores, such as Nicotinamide adenine dinucleotide (NADH) and Flavin adenine dinucleotide (FAD), the differences in signal strength between healthy and tumor tissue are weak and limit their potential for surgical guidance [207,208]. Exogenous probes can provide stronger signals and greater contrast by using molecules and nanoparticles that exhibit high quantum yields and tunable absorption/emission profiles, and modifying them to target tumors [209]. Dye molecules are among the most widely used probes because their chemical structure can be modified to tune their spectral wavelength, they efficiently penetrate tumors via diffusion, and dyes can be conjugated to antibodies and peptides for targeting [210]. However, dye molecules can photodegrade via oxidation under prolonged exposure to their excitation wavelength, irreversibly altering their structure and reducing their signal strength. Nanoparticle probes, such as ultra-small gold nanoparticles, offer an alternative approach and exhibit greater photostability than dye molecules. These ultra-small nanoparticles are well-defined aggregates of gold atoms that bridge the optical properties of single atoms and larger plasmonic nanoparticles. To reflect this, they are often termed nanoclusters rather than nanoparticles [165]. Nanoclusters typically contain fewer than 100 gold atoms and have a core diameter below 3 nm. Unlike larger plasmonic nanoparticles, they have discrete molecular-like electronic states that can be tuned by controlling the size, doping, and surface ligands of the nanocluster [211]. Similar to larger nanoparticles, non-targeted ultra-small nanoparticles accumulate in tumors via the EPR effect, making them attractive photostable contrast agents for photoluminescent imaging [212]. The small size of nanoclusters means they are renally cleared following systemic administration, making them attractive contrast agents that, unlike larger metallic NPs, do not persist in the organs of the reticuloendothelial system months after administration [213,214]. Furthermore, nanoclusters have been demonstrated to exhibit tunable photoluminescent emission across the visible to NIR-II, making them uniquely versatile contrast agents [215].

Glutathione-capped gold nanoclusters (AuNCs) have been assembled into chitosan/Gd^3+^-coordinated supraclusters that are tuned to emit in the NIR-II and target specific organs following intravenous injection [216]. AuNCs (~1.5 nm) were assembled with chitosan via electrostatic interactions and Gd^3 +^ was added incrementally as a chelating ion to create three supracluster types: Au-CS (glutathione only, d = 30 nm), AuGd-Low (low concentration Gd^3+^, 65 nm), and AuGd-High (high concentration Gd^3+^, 200 nm). The concentration of Gd^3 +^ used to assemble the supraclusters determined their size and biostability. In turn, these physical properties determined the organotropic fate of the supraclusters. The *in vivo* pharmacokinetics, urinary clearance, and biodistribution of each supracluster were tracked by NIR-II photoluminescence, followed by endpoint analysis with ICP-MS. T_1_-weighted MRI using Gd contrast was used to monitor biodistribution. NIR-II photoluminescence imaging performance was compared for AuNCs, AuGd/L, and AuGd/H in healthy and tumor-bearing mice. Supracluster size strongly influenced organ-specific accumulation. Au-CS assemblies readily disassembled into nanoclusters that preferentially accumulated in the kidneys, as expected for renal clearance, whereas AuGd-Low supraclusters were more stable and accumulated predominantly in the liver, consistent with the behavior of larger particles [213]. Surprisingly, AuGd-High supraclusters exhibited greater stability and distributed to both the liver and lungs, reaching ~13% ID/g in the lungs at 3 h post-injection; lung signal then decreased over 24 h, consistent with clearance via progressive disassembly (Fig. 6C-D). In mice bearing orthotopic lung tumors, AuGd-high produced strong lung NIR-II contrast, with a signal to background ratio of 2.7 at 10 h, co-localizing with tumor regions. The authors hypothesized that stronger Gd^3 +^ co-ordination increased biostability and serum-protein adsorption, yielding micron-scale aggregates that transiently trap in lung microvasculature, and that red blood cell “hitchhiking” may contribute. This imaging approach has the advantage of tuning nanoparticle biodistribution using the physical properties of the probes and utilizing an imaging agent that is renally clearable, unlike larger plasmonic NPs used for SERS-based imaging

Nanoclusters have also found applications in pathology. There is currently low diagnostic accuracy and limited spatial resolution for breast cancer molecular typing methods. One study aimed to address this by developing praseodymium(Pr)-doped gold nanoclusters (Au24Pr1) that emit in the NIR-II window and are directly conjugated to anti-estrogen receptor (ER), anti-progestersone receptor (PR), and HER2 mAbs [217]. The probes enabled accurate molecular subtyping and 3D visualization in clinical breast cancer sections and biopsies with higher signal-to-background ratios than conventional NIR-I imaging. Au24Pr1 were fabricated by single-atom Pr substitution of gold nanoclusters to boost NIR-II brightness by a factor of 3.9 and improve photostability. TEM images showed that the nanoclusters had an average diameter of approximately 2 nm, while DLS measurements indicated hydrodynamic diameters ranging from 2.7 to 3.1 nm. The zeta potential was measured to be −36.5 mV. Covalent conjugation of primary mAbs for ER, PR, and HER2 to the nanoclusters increased hydrodynamic size to ~28 nm. The authors imaged 12 clinical sections from breast cancer subtypes Luminal A, Luminal B, HER2-enriched, and triple-negative, plus intact biopsy cores, using NIR-II fluorescence microscopy and a home-built NIR-II light-sheet microscope. Excitation wavelengths of 680, 730, and 808 nm were evaluated, with emission collected from 850 to 1700 nm; optimal performance was achieved using 730 nm excitation with emission centered near 1100 nm. Quantification included subtype-specific expression maps and signal-to-background ratio analyses versus NIR-I imaging. Pr single-atom doping enhanced NIR-II emission at 1100 nm, improved stability, and eliminated photobleaching during 60 min of laser exposure. NIR-II light-sheet microscopy achieved 500 μm penetration and 2.1 × 2.1 × 3.5 μm voxel resolution at 50 fps in tissue. Relative to NIR-I, the background signal decreased, and the signal-to-background ratio rose by a factor of 4. This enabled clear mapping of the biomarkers ER, PR, and HER2 giving robust discrimination of Luminal A, Luminal B, HER2-enriched, and triple-negative cancer subtypes. NIR-II results were consistent with immunohistochemistry. Advantages of this approach are the high signal-to-background ratio, the large field of view achieved during 3D imaging and rapid subtype discrimination without requiring the input of highly trained pathologists.

In addition to imaging agents, the optical properties of nanoclusters can be leveraged to add a cancer therapy modality by using them as photothermal therapy agents. Baghdasaryan *et al*. engineered ~2 nm Au25 gold molecular clusters with a zwitterionic phosphorylcholine shell (AuPC) to enable uniform tumoral distribution and facilitate NIR-II/short-wave infrared imaging and photothermal therapy of breast cancer [218]. Upon intratumoral injection, AuPC distributed homogeneously through tumor interstitial fluid space, providing margin guidance for resection and a uniform absorption of light and heat generation during photothermal therapy. *In vivo*, NIR-IIb (1500–1700 nm) quantum dot-Annexin V probes were used to detect apoptosis following photothermal therapy. The platform showed high biocompatibility and rapid renal clearance, making it of translational interest. Glutathione-coated Au25 clusters were conjugated to 4-aminophenyl-phosphorylcholine using carbodiimide crosslinker chemistry to form AuPC. BALB/c mice bearing 4T1 tumors received intratumoral injections of 300 μg in 20–30 μL PBS, followed by wide-field NIR-II imaging for margin delineation with excitation at 808 nm and collection at > 1100 nm using a long-pass filter. Control experiments were run with glutathione-coated Au25 and indocyanine green. Au25 clusters concentration in the tumor interstitial fluid was quantified by resection, purification by high-speed centrifugation, and NIR-II measurement of extracts. For photothermal therapy, tumors were irradiated at 808 nm (300 mW cm^−2^, 30 min) and thermal profiles were recorded with a thermal imaging camera. Cell death mechanisms were probed *in vivo* with quantum dot (QD)-P3-Annexin V and compared to non-targeted QD-P3 using NIR-IIb imaging. Apoptosis was further quantified by flow cytometry using an Annexin V-fluorescein isothiocyanate (FITC)/propidium iodide (PI) double-stain 24 h post-photothermal therapy, along with a TUNEL on cryosections of tumor tissue. Intratumoral administration of AuPC produced uniform intratumoral fluorescence with a tumor-to-surrounding background tissue ratio (TBR) of ~ 50, enabling complete resection with non-excessive tissue removal. In contrast, control experiments with the clinically approved fluorescent contrast agent indocyanine green yielded a TBR of ~ 4. Probe localization in the interstitial fluid resulted in a spatially homogeneous signal. As a photothermal agent, AuPC heated tumors to ~60 °C at 808 nm excitation, triggering tumor regression in all but 1 of 18 mice. A 60-day survival of 94.4% was measured following photothermal therapy. Post-photothermal therapy, QD-P3-Annexin V imaging highlighted apoptotic regions with tumor-to-normal tissue (TNR) ratios of 20, indicating a specific accumulation in apoptotic tumor tissue. These results were corroborated by flow cytometry and TUNEL staining. This study demonstrates the homogeneous labeling and heating of breast cancer models with low off-target staining and impressive survival rates following treatment. A primary advantage of this study is its use of ultra-small nanoclusters that are renally clearable, unlike larger (> 5 nm) noble metal NPs. However, a major limitation is the undefined nature of the background and normal tissue used to calculate TNR and TBR, limiting comparisons of AuPC with tumor margin delineation probes reported in other published works.

## Conclusion and future perspectives

The cancer diagnostics market has undergone a paradigm shift driven by nanotechnology over the past ten years, enabling the detection and visualization of disease at the molecular level. This review considered recent advances in nano-engineered metals as sensing and imaging tools, which have matured from laboratory-scale prototypes to practical applications. Section 2 highlighted the development of innovative nanomaterial-based sensing platforms, including electrochemical, optical, ECL, PEC, SPR, SERS, biofuel-cell-driven, two-signal, CHA/HCR-assisted, ratiometric, and CRISPR-based methods, while Section 3 described recent advances in nanotechnology-boosted cancer imaging tools, including MRI, Raman/SERS, fluorescence, CD, PA, multimodal nanoprobe platform development, and *in vivo* tumor visualization. Taken together, these advances indicate a paradigm shift in cancer diagnostics.

Biomarker diagnosis in cancer is shifting away from bulk biochemistry toward nanoscopic interrogation. The nanotechnology-assisted sensing presented LODs previously unattainable, with capabilities for multiplexing, high reproducibility, biological target specificity, even in complex biological fluids, and non- or minimal invasiveness. Biosensor platforms for detecting circulating cancer signatures, such as EVs, exosomes, miRNA, CTCs, circRNA, oncogenes, mutation signatures of ctDNA, metabolic signatures of cancer, telomerase activity, DNA methylation profiles, and HSP70 protein, appear highly promising for early-stage cancer diagnosis, patient stratification for different treatments, and monitoring for recurrence. These platforms could detect low quantities of miRNA, individual CTCs, far surpassing what has been attainable by traditional biochemical and immunoassays. These nanoscale sensing platforms have made significant contributions through the use of metal nanomaterials in combination with MOFs, magnetic nanobeads, MXene hybrids, COFs, MOF-derived nanozymes, hollow nanostructures, and plasmonic interfaces. They offer high tunable bioaffinity, low nonspecific fouling, and efficient transduction.

In addition, breakthroughs in imaging nanotechnology (Section 3) have increasingly transformed cancer imaging from anatomical and functional visualization toward functionally responsive, molecularly informed readouts. Nanosensors employed to enhance MRI contrast, SERS-based molecular imaging methods, fluorescence imaging techniques, circular dichroism bioimaging techniques, and photoacoustic scanning methods can deliver deep-tissue resolution and highly sensitive and specific contrast at the cellular level. Enzymatically responsive intelligent NPs, such as supraparticles, SERS probes isolated with a shell, plasmonic nanotags, and magnetic nanocomplexes, enable the noninvasive imaging of tumor microenvironment biomarkers, including enzymatic activity, proteolysis, peptide cleavage, metabolic concentration gradients, and receptors. These approaches deliver molecular and spatial information that cannot be captured through serum biomarkers alone and open the door to real-time delineation of tumor boundaries during surgical or interventional procedures.

While sensing and imaging have developed in parallel, one of the most important findings of this review is their natural integration. Nanotechnology has enabled the creation of sensing components that are, simultaneously, imaging agents, thereby providing a dual-function diagnostic platform. SERS nanoprobes, which can detect circulating nucleic acids, can further enable visualization of cells via fluorescence. The importance of multiplexed NPs in increasing the confidence level of detection parameters, based on both molecular recognition and tumor visualization, cannot be ignored. Future directions for both sensing and imaging technologies will enable nano-diagnostic systems that can evaluate biomarker levels while providing visualization.

Although great progress has been made, the practical implementation of these nanotechnologies for widespread medical use remains challenging. With regard to sensing-based systems, the greatest challenge is validation for medical use. Almost all biosensors described thus far are validated for laboratory or restricted, controlled patient samples and simulated biologic environments, rather than for more comprehensive patient validation. Comprehensive validation of patient groups is ultimately required to demonstrate reliability, reduce false-positives, and determine the medical relevance of the operating threshold boundaries. Standardization of the electrode surface, nanomaterial preparation, electrodeposition, recognition element stability, and sample preparation methods would pose potential issues of reduced specificity for practical approval, although great improvements are needed here. In addition, the biochemical complexity of the cancer cell microenvironment complicates biomarker expression patterns due to biological variability.

In imaging nanotechnology, translation to the clinic remains constrained by biosafety and regulatory considerations. NPs intended for *in vivo* imaging, including magnetic supraparticles, heavy-metal SERS tags, and non-biodegradable core–shell nanostructures, must demonstrate well-defined biodistribution, predictable renal or hepatic clearance, minimal long-term tissue accumulation, and negligible immunogenicity to meet regulatory expectations. Potential toxicity remains a major barrier to translation from proof-of-concept studies to first-in-human trials. Accordingly, current nanotechnology design strategies increasingly prioritize biodegradable materials, organic polymeric matrices, renal-clearable ultrasmall constructs, stimulus-responsive disassembly, and metabolizable organic domains over persistent inorganic architectures. In parallel, successful clinical translation requires seamless integration with existing radiologic infrastructure; imaging agents must be compatible with standard MRI, CT, PET, or optical imaging platforms without necessitating hardware modification or changes to image reconstruction workflows.

Looking ahead, the trajectory of cancer diagnostics over the next decade will be shaped by several disruptive technologies, including theranostics, molecular Imaging, point-of-care nanodevices, AI-driven multi-omics, and implantable diagnostic microelectronics. Among these, nanotheranostics — nanomaterials that integrate diagnostic and therapeutic functions — hold particularly strong clinical promise. As discussed in this review, such platforms enable the simultaneous detection of cancer-associated biomarkers and the delivery of therapeutic payloads, including small-molecule drugs, radioisotopes, or photothermal agents, thereby supporting real-time assessment of treatment response. In parallel, live molecular imaging is expected to advance through nanometer-scale precision in contrast agent design. Emerging classes of activatable and functionally responsive imaging probes are shifting molecular imaging from passive visualization toward dynamic interrogation of underlying biochemical processes. These advances are expected to support increasingly sophisticated image-guided interventions and more refined assessment of therapeutic distribution and response within tumors. Looking ahead, integrated sensing–imaging strategies could enable real-time insights into treatment efficacy and cellular response in situ, facilitating more adaptive and personalized therapeutic decision-making. Secondly, the nanodiagnostic kit at the point of care will extend cancer testing beyond hospitals. This expansion will be driven by miniaturized micro/nanoelectrode chips, paper-based sensors, lateral-flow immunoassays using nanoprobes, and smartphone-based scanners. These technologies will enable a dramatic expansion of early cancer testing from hospitals to clinics, pharmacies, and households. In addition, multi-omics integration, the merging of proteomics, genomics, transcriptomics, lipidomics, and metabolomics, can compensate for the shortcomings of single-biomarker techniques. The nanotechnology platform enables multi-analyte sensing at the picomolar level through its architecture, which appears to support AI algorithms that merge these data types into predictions for the final diagnosis. Finally, implantable nanosensors are a frontier area where sensing and imaging can be integrated with continuous surveillance. Subcutaneous electrochemical chips, microneedle sensor arrays, flexible nanosheets, and transcutaneous electrofluidic patches can continuously quantify tumor marker levels for weeks or months. These can detect relapse or the onset of metastases before the appearance of symptoms of a malignant disease that could lead to death.

Highly sensitive biosensing has shown early detection of molecular alterations long before anatomical changes can be demonstrated in radiological imaging, while advanced imaging has shown spatial resolution and understanding that are not feasible in standalone biomarker assessments. Together, these capabilities constitute a powerful modality for early detection, precise staging and evaluation, and accurate treatment and follow-through evaluation. The technological advancements presented in this review indicate that cancer diagnostics will soon enter an era of nanometer-scale accuracy and sophistication, wherein diseases will be assessed at nanometer dimensions as they begin their molecular course, visualized in living tissue in real time, and interpreted computationally and synergistically on a single platform. Technological and scientific fields continue to converge, and nanotechnology in cancer diagnostics and imaging solutions will not only enhance cancer diagnostics; it will redefine its architecture, with timelines for diagnostic procedures expedited, death rates reduced, cost and accessibility achieved, and access to precision medicine made global.

## Figures and Tables

**Table 1:** Summary of recent nanomaterial-based sensing platforms developed for cancer diagnostics.

Type of cancer	Inorganic materials in the nanotag's structure	Size of nanomaterials	Shape	Nanotag (s) info	Sample origin	Modality	Signal Transducer (s)	Target Biomarker	Detection range	LOD	Ref.
Breast cancer	Core-Shell Magnetic (Fe_3_O_4_@PDA) NPs	500 nm	Spherical	Conjugated with an aptamer	Human serum samples	Electrochemical (DPV)	ITO electrode	Breast cancer cell-derived EVs	700–1000000 particles mL^−1^	220 particles mL^−1^	[54]
Breast cancer	Au NPs	- ♣	Spherical	Conjugated with DNA	Human serum samples	Electrochemical (DPV)	Carbon cloth electrode	miRNA-451/miRNA-145	1–100 fM	miRNA-451: 0.240 fM/miRNA-145: 0.317 fM	[60]
Breast cancer	Co@Co_3_O_4_/CoO@Co_3_O_4_ nanocomposites	-	Spherical	Interaction with hyaluronic acid	Human serum samples	Electrochemical (DPV)	ITO electrode	CD44	1 × 10^−5^ - 1 × 1000 ng mL^−1^	0.619 × 10^−5^ ng mL^−1^	[145]
Breast cancer	Ag NPs	19.88 nm	Spherical	Conjugated with a peptide	Human serum samples	Electrochemical (DPV)	GE	HER2	0.05–2 ng mL^−1^	0.05 pg mL^−1^	[146]
Breast cancer	MB@COFs nanocomposite	425 nm	Spherical	Conjugated with an aptamer	Human serum samples	Electrochemical (SWV)	GCE	CTCs (MCF-7 cells)	10–1 × 10^7^ cells mL^−1^	1 cells mL^−1^	[63]
Breast cancer	SiO_2_ NPs/ Magnetic beads	SiO_2_ NPs: 100 nm/ Magnetic beads: 300 nm	Spherical	Conjugated with an antibody	Human serum samples	Electrochemical (SWV)	SPCE	HER2/ ER/ Ki67/ PR	Up to 1000 pg mL^−1^	HER2: 2 pg mL^−1^/ ER: 1.8 pg mL^−1^/ Ki67: 10.36 pg mL^−1^/ PR: 1.33 pg mL^−1^	[147]
Breast cancer	Au@CeO_2_@CuS nanocomposite	-	Rod-like	Conjugated with DNA	Human serum samples	Electrochemical (chronoamperometry (CA))	GCE	Breast Cancer Gene 1 (BRCA1)	0.1 pM - 10 nM	0.02 fM	[148]
Breast cancer	ZnNi-MOF nanocomposite	300 nm	Wrinkle-like spherical	Conjugated with an antibody	Human serum samples	ECL	GCE	CA15–3	0.0005–50 mL^−1^	5.75 × 10^−5^ mL^−1^	[149]
Breast cancer	Magnetic beads	-	Spherical	Conjugated with DNA	Human serum samples	ECL	GCE	CEA/ BRCA1	CEA: 10 fg mL^−1^ - 1 μg mL^−1^/ BRCA1: 0.1 pM - 0.1 μM	CEA: 6.79 fg mL^−1^/ BRCA1: 46.25 fM	[150]
Breast cancer	PtCo@rGO nanozyme	-	Cubic	Conjugated with DNA	Human serum samples	ECL	GCE	MCF-7 cells	2–10000 cells mL^−1^	1 cells mL^−1^	[151]
Breast cancer	Magnetic beads	-	Spherical	Conjugated with DNA	Whole blood samples	Fluorescent/ Electrochemical (DPV)	Fluorescent: A tube/ Electrochemical: SPCE	Long noncoding RNAs (lncRNAs)	Fluorescent: 1 pM - 100 nM/ Electrochemical: 100 fM - 1 nM	Fluorescent: (HOX transcript antisense intergenic RNA (HOTAIR): 0.43 pM / Metastasis-associated lung adenocarcinoma transcript 1 (MALAT1): 0.77 pM) / Electrochemical: (HOX transcript antisense intergenic RNA (HOTAIR): 15.2 fM / Metastasis-associated lung adenocarcinoma transcript 1 (MALAT1): 6.3 fM)	[152]
Breast cancer	Magnetic beads	850 nm	Spherical	Conjugated with an aptamer	Human serum samples	Fluorescent/ Visual	CdTe quantum dots on a surface	MCF-7-related exosomes	Fluorescent: 7.9 × 10^4^ - 2.2 × 10^6^ particles μL^−1^/ Visual: up to 300 particles mL^−1^	Fluorescent: 1.1 particles μL^−1^/ Visual: 300 particles mL^−1^	[57]
Breast cancer	MOF-Fe_3_O_4_ nanocomposite	53.7 nm	Rod-like nanostructures	Conjugated with DNA	Human serum samples	Colorimetric (ultraviolet–visible spectroscopy (UV-Vis))	A lab tube	PD-L1	Circulating PD-L1: 0–10 ng/mL; Exosomal PD-L1: 1 pg mL^−1^ - 5 ng mL^−1^	Circulating PD-L1: 5 pg mL^−1^; Exosomal PD-L1: 1 pg mL^−1^	[153]
Lung cancer	Magnetic beads	2.8 μm	Spherical	Conjugated with an antibody	Human serum samples	Fluorescent	A glass	CEA	1 fg mL^−1^ - 10 ng mL^−1^	- *	[154]
Lung cancer	Magnetic beads	-	Spherical	Conjugated with streptavidin	Human serum samples	Fluorescent/ Electrochemical (DPV)	GCE	ctDNA	Fluorescent: 1 pM - 1 μM/ Electrochemical (DPV): 10 fM - 1 nM	Fluorescent: 14.36 fM/ Electrochemical (DPV): 372 aM	[102]
Lung cancer	Magnetic beads	-	Spherical	Conjugated with DNA	Human serum samples	ECL	GCE	miRNA-622	10 fM - 500 nM	1.09 fM	[155]
Lung cancer	Fe_3_O_4_@Au NPs nanocomposite/ Au@MBA@Ag NPs nanocomposite/ Au@TFMBA@Ag NPs nanocomposite	~ 50 nm	Spherical	Interaction with DNA and antibody	FFPE extracted DNA	SERS	A glass contains PBS buffer	SHOX2 gene/ RASSF1A gene	10 pM - 100 nM	SHOX2: 0.52 pM; RASSF1A: 0.66 pM	[93]
Lung cancer	Fe_3_O_4_-SiO_2_-TiO_2_ nanocomposite	-	Not presented	Conjugated with an aptamer	Real sample evaluation not performed.	Colorimetric (UV-Vis)	NA	PD-L1	1000–10000000000 Evs mL^−1^	360 Evs mL^−1^	[156]
NSCLC	N-CQDs/ AuNPs	Au NPs: 9–12 nm	Au NPs: sphere/ N-CQDs: dot	Conjugated with an aptamer	Peripheral blood samples	Electrochemical (SWV)	SPCE	CTCs (H1299 and A549 cells)	Up to 500 ng mL^−1^	2 ng mL^−1^	[90]
NSCLC	Silica NPs	-	Spherical	Conjugated with DNA	Real sample evaluation not performed.	Electrochemical (DPV)	GCE	DNA sequence of gastric cancer	1 fM - 1 nM	0.13 fM	[157]
NSCLC	His@ZIF-8/Fe-TCPP nanocomposite	-	Cube	Conjugated with DNA	Human serum samples	ECL	GCE	ctDNA	1 fM - 100 nM	0.35 fM	[158]
HCC	GO/ Au NPs	Au NPs: 20 nm	GO: 2D sheets/ Au NPs: Spherical	Conjugated with an antibody	Human serum samples	Electrochemical (DPV)	GCE	AFP	10 fg mL^−1^ - 10 ng mL^−1^	3.3 fg mL^−1^	[159]
HCC	Lens culinaris agglutinin (LCA)@AgNPs nanomaterial	-	Spherical	Conjugated with protein	Human serum samples	Electrochemical (DPV)	GCE	AFP	5–150 ng mL^−1^	69 pg mL^−1^	[160]
HCC	Fe_3_O_4_@Au nanocomposite	200–350 nm	Spherical	Conjugated with an aptamer	Human serum samples	Electrochemical (EIS)	GCE	HSP70	10 pg mL^−1^ - 200 ng mL^−1^	0.53 pg mL^−1^	[79]
HCC	CdSe QD-NaYF4:Yb,Er UCNPs hybrid nanomaterial	10 nm	Irregular spherical shapes	Conjugated with DNA	Human serum samples	Photoelectrochemical (PEC)	SPE	ctDNA	400 aM - 200 pM	2.32 aM	[161]
Liver cancer	Dendritic mesoporous silica nanoscaffold (DMSN)-Au NPs-Ag nanocomposite	370 nm	Spherical	Conjugated with an antibody	Human serum samples	Electrochemical (DPV)	SPEs	AFP	Up to 500 ng mL^−1^	0.85 ng mL^−1^	[81]
Liver cancer	Au NPs	-	Spherical	In interaction with concanavalin	Human serum samples	Electrochemical (DPV)	GCE	AFP	10 fg mL^−1^ - 10 ng mL^−1^	3.41 fg mL^−1^	[162]
CRC	Au@Ag nanomaterial	37 nm	Spherical	Conjugated with an aptamer	Human serum samples	SERS	A glass	TNF-α	0.1–100000 pg mL^−1^	0.1 pg mL^−1^	[163]
CRC	Reduced titanium nanoclusters	Lattice fringe spacing: 0.264 nm	Spherical	Conjugated with DNA	Colorectal and paracancerous tissues	ECL	GCE	miRNA-128	1 fM - 1 nM	0.17 fM	[72]
Oral cancer overexpressed 1	Ag NPs/ GO@UiO-66 nanocomposite	Ag NPs: 6.8 nm/ UiO-66: 100 nm	Ag NPs: sphere/ UiO-66: octahedral	Conjugated with DNA	Saliva samples	Electrochemical (SWV)	GCE	DNA sequence of oral cancer overexpressed 1	0.01 fM - 1 pM	0.28 aM	[85]
Ovarian cancer	AuNPs@2DCOFBTT-DGMH nanocomposite	-	Irregular shapes	Conjugated with an antibody	Real sample evaluation not performed.	Electrochemical (DPV)	GCE	CA-125	0.00027–100 mL^−1^	0.089 mU mL^−1^	[164]
Bladder cancer	Au NPs	-	Spherical	Conjugated with streptavidin	Urine samples	SPR	CM5 SPR chip	miRNA-183/ miRNA-155	Up to 10 nM	0.57 pM	[69]
Leukemia	Magnetic microbeads	~ 1.5 μm	Spherical	Conjugated with DNA	Human serum samples	Amperometry (i-t)	Magnetic GCE	DNA sequence of Leukemia	500 aM - 10 pM	100 aM	[165]
Gastric cancer	Fe_3_O_4_ NPs	-	Spherical	None	Human tissue	NPELDI-MS	Electron multiplier detector	Metabolic fingerprinting	-	5.6–11.2 pM for panel of standard small metabolites	[138]
Gastric, pancreatic, and colorectal cancer	Fe_3_O_4_ NPs	-	Spherical	None	Human serum	NPELDI-MS	Electron multiplier detector	Metabolic fingerprinting	Metabolite -specific, e.g., 10–2,000 μM for phenylalanine	0.07–292.13 μM for different metabolite standards	[140]
Ovarian cancer	Fe_3_O_4_ NPs	-	Spherical	None	Human serum	NPELDI-MS	Electron multiplier detector	Metabolic biomarker panel	-	-	[166]
Other Cancers	Au NPs	20 nm	Spherical	In interaction with PNA and telomerase	Urine biopsy	Electrochemical (DPV)	GE	PNA in urine biopsy	- ♠	176 nM	[103]
Other Cancers	BQD/NH_2_-MXene@Au NPs nanohybrids	Au NPs: 10 nm/ UiO-66: 100 nm	Spherical	Conjugated with an antibody	Human serum samples	Electrochemical (DPV)	GCE	Midkine, a neurotrophic growth factor	5 fg mL^−1^ - 100 ng mL^−1^	1.62 fg mL^−1^	[107]
Other Cancers	Co-HOF nanomaterial	Primary synthesis solution particles: 1.17 μm	Rod-like nanomaterial	Conjugated with an antibody	Human blood samples	Electrochemical (DPV)	GCE	CEA	0.001–50 ng mL^−1^	0.22 pg mL^−1^	[132]
Other Cancers	Au NPs@COF	-	Irregular spherical shapes	Conjugated with an antibody	Human serum samples	Electrochemical (DPV)	GCE	CA-125	0.01–100 mL^−1^	0.0067 mL^−1^	[167]
Other Cancers	Au NPs/ GODs	AuNPs: 0.25 nm/ GODs: 0.47 nm	AuNPs: Spherical/ GODs: Dots	Conjugated with DNA	Human serum samples	Electrochemical (DPV)	Carbon cloth electrode	miRNA–21	0.01 fM - 0.1 nM	0.0028 fM	[168]
Other Cancers	NiCo@Fc-MWCNTs-LDH nanocomposite	-	Brush-like structure	Conjugated with an aptamer	Human serum samples	Electrochemical (DPV)	GCE	EVs	1.6 × 10^2^ - 1.6 × 10^6^ particles mL^−1^	13.79 particles mL^−1^	[169]
Other Cancers	Magnetic beads	122 nm	Spherical	Encapsulated with liposomes	MCF-10A (normal breast cell), MCF-7 (breast cancer cell), and MDA-MB-231 (breast cancer cell)	Electrochemical (SWV)	Cucurbituril [7] electrode	MMP-2	50 fg mL^−1^ - 10 ng mL^−1^	13.3 fg mL^−1^	[112]
Other Cancers	ZnO@CuS nanocomposite	184 nm	Spherical	Conjugated with DNA	Human serum samples	Electrochemical (SWV)	GCE	miRNA-155	0.5 fM - 5 nM	0.11 fM	[170]
Other Cancers	Lipid NPs (LNPs)	-	Spherical	Conjugated with DNA	- ♥	Electrochemical (SWV)	Screen-printed gold electrodes	miRNA-218	Up to 800 nM	0.45 nM	[171]
Other Cancers	Magnetic beads	-	Spherical	Interaction with the aptamer and the antibody	Human serum samples	Electrochemical (Chronocoulometry)	Nitrocellulose-modified electrode	HER-2/neu(+)	0.1 fM - 1 pM	0.1 fM	[172]
Other Cancers	Au@CuS nanocomposite	300 nm × 500 nm	Irregular shapes	Conjugated with an antibody	Human serum samples	Electrochemical (CA)	GCE	CEA	0.01 pg mL^−1^ - 0.5 ng mL^−1^	3.8 fg mL^−1^	[173]
Other Cancers	Magnetic beads	-	Spherical	Conjugated with DNA	Human serum samples	ECL	GCE	m6A-RNA/ m5C-RNA	m6A-RNA:10^−13^ - 10^−6^ M ; m5C-RNA: 10^−14^ - 10^−7^ M	m6A-RNA: 213 aM; m5C-RNA: 2.08 fM	[174]
Other Cancers	Magnetic beads/ Au NPs	Au NPs: 20 nm/ Magnetic beads: not mentioned	Spherical	Conjugated with DNA	Human serum samples	ECL	ITO electrode	miRNA-21	10 fM - 100 nM	0.48 fM	[175]
Other Cancers	Magnetic carbon nanotubes (MCNTs)	-	Tubal	Conjugated with DNA	Human serum samples	ECL	SPCE	miRNA-21/ miRNA-183	0.1–1000 fM	miRNA-21: 0.38 fM; miRNA-183: 0.49 fM	[176]
Other Cancers	Au@Cu_2_O nanocomposite	80 nm	Irregular spherical shapes	Conjugated with an aptamer	Human serum samples	PEC	ITO electrode	CEA	0.1 pg mL^−1^ - 10 ng mL^−1^	0.03 pg mL^−1^	[177]
Other Cancers	Fe_3_O_4_@SiO_2_ nanocomposite	-	Spherical	Conjugated with DNA	Whole blood samples	PEC	ITO electrode	LncRNAs	10^2^ - 10^7^ aM	lncRNA HOTAIR: 25.5 aM/ lncRNA MALAT1: 53.1 aM	[178]
Other Cancers	Fe-MOF nanocarrier	28 nm	Octahedron	Conjugated with DNA	Human serum samples	Electrochemical (DPV)/ Fluorescent/ UV–vis	Electrochemical (DPV): SPE/ Fluorescent: A tube/ UV–vis: A tube	miRNA-155	Electrochemical: 500 aM - 100 pM/ Fluorescent: 5 fM - 100 pM/ UV–vis: 50 fM - 100 pM	Electrochemical: 0.34 fM/ Fluorescent: 2.97 fM/ UV–vis: 20.75 fM	[179]
Other Cancers	Hyaluronic acid-ZIF-8 nanocomposite	200 nm	Octahedron	Conjugated with DNA	HepG2 cells	Electrochemical (Enzymatic biofuel cells)	Fluorine-doped Tin oxide electrode	CTCs	50–10^6^ cells mL^−1^	3 cells mL^−1^	[180]
Other Cancers	Cholesterol-modified magnetic beads	-	Spherical	Conjugated with DNA	Plasma of colon and gastric cancers	Fluorescent	A glass	Tumor-derived EVs PD-L1	10–1000000 particles μL^−1^	7.5 particles μL^−1^	[181]
Other Cancers	Magnetic beads	-	Spherical	Conjugated with protein	Human serum samples	Fluorescent	Solid-Phase Interface-Mediated	Flap endonuclease 1	1 × 10^−5^ - 3 × 10^−3^ U μL^−1^	2.53 × 10^−6^ U μL^−1^	[182]
Other Cancers	Au NPs	10 nm	Spherical	Conjugated with DNA	Cells from breast cancer patient tissues	Fluorescent	A tube	CircRNAs	1 × 10^−8^ - 0.1 nM	CircFOXO3: 8.34 aM/ CircMTO1: 9.84 aM	[183]
Other Cancers	Cobalt boride nanosheet (CoB NS)	50 nm	Square or round shapes	Conjugated with DNA	Tumor cell H1975	Fluorescent	3D-pop-up paper-based signal interface	ctDNA	200–1500 pM	79.88 pM	[184]
Other Cancers	Au@4MBN@Au nanocomposite	43 nm	Spherical	Conjugated with DNA	Real sample evaluation not performed.	SERS	Quartz sheets	CircRNAs	1 pM - 100 μM	0.043 pM	[128]
Other Cancers	Ag NPs@4-MBA	-	Spherical	Conjugated with an antibody	Human serum samples	SERS	Au nanorod film on a microbowl array	CA-125	1–5000 mL^−1^	1 mL^−1^	[136]
Other Cancers	Chiral MnO_2_ SPs	150 nm	Spherical	Conjugated with a peptide	Mice urine	CD/ MRI	CD and MRI signal interfaces	Metalloproteinase-9	0.01–10 ng mL^−1^	CD: 0.0054 ng mL^−1^/ MRI: 0.0062 ng mL^−1^	[117]
Other Cancers	Fe_3_O_4_ NPs	200 nm	Spherical	In interaction with DNA and an aptamer	A549 cells	Low-field nuclear magnetic resonance	A tube	CTCs	10–1 × 10^6^ cells mL^−1^	6 cells mL^−1^	[185]
Other Cancers	Ag@Magnetic NPs nanocomposite	143 nm	Spherical	Conjugated with an aptamer	Human serum samples	Colorimetric (UV-Vis)	A lab tube	PD-L1^+^ exosomes/ MUC1	10^3^ - 10^9^ particles mL^−1^	37.2 particles mL^−1^	[186]

♣: Size of nanomaterials not mentioned

*: LOD not mentioned

♠: Detection range not mentioned

♥: Real sample application not mentioned.

**Table 2:** Summary of recent nanomaterial-based sensing platforms developed for cancer imaging.

Cancer type	NPs material	Size (nm)	Shape	Imaging modality	Testing model	Targeting	Therapeutic component	Reference
Brain glioblastoma	Au, Ag	39.4 (±3.7) x 57.5 (±5.0)	Rod-in-cuboid	SERS	Orthotopic U87-MG (BALB/c-nu)	Homotypic targeting via glioblastoma (U87-MG)-derived cell membrane	No	[193]
Breast	Au, Pr	2	Nanocluster	NIR-II photoluminescence	Excised patient tumor samples	Estrogen receptor (ER), progesterone receptor (PR), human epidermal growth factor receptor 2 (HER2)	No	[219]
Colon adenocarcinoma	Gd-Zn-Cu-In-S/ZnS quantum dots	5	Sphere	CT, MRI, photoluminescence	Subcutaneous HT-29 (Immunosuppressed C57BL/6)	Epithelial cell adhesion molecule (EpCAM)	Chemotherapy (epirubicin)	[220]
Breast	Au	3	Nanocluster	NIR-II photoluminescence	Subcutaneous 4T1 (BALB/c)	Intratumoral injection	No	[218]
Pancreatic ductal adenocarcinoma	Au	38.06 ± 6.2	Sphere	Fluorescence, photoacoustic	Subcutaneous Panc02 (C57BL/6)	Cathepsin E	Photothermal therapy + IDO1 inhibitor	[221]
Lymphoma	Au	50 × 8	Rod	Ultrasound, photoacoustic	Subcutaneous EL4 (C57BL/6J)	Ovalbumin (OVA)	No	[204]
Breast	Au	40	Sphere	Photoacoustic	Subcutaneous MDA-MB-231 (NU/J)	Integrin αvβ3	No	[222]
Breast	Au	40 × 17	Rod	SERS, photoacoustic	Subcutaneous 4T1 (BALB/c)	Homologous targeting (4T1 cancer cell membranes)	Photothermal therapy + photodynamic therapy	[223]
Breast	Au, Cu(II)	2.87 ± 0.52	Sphere	CT	Subcutaneous 4T1 (BALB/c-nu)	Folic acid	Photodynamic therapy	[224]
Breast	Gd, Ti	224.7 ± 5.8	Sphere	MRI	Subcutaneous 4T1 (C57BL/6)	Folic acid	ROS production when combined with^18^FDG	[225]
Cervical	Au		Rod	SERS	Subcutaneous HeLA (BALB/c-nu)	No - intratumoral injection	No	[199]
Breast	Au, Ag	51.6 (±3.8)	Cube	SERS	Subcutaneous 4T1 (BALB/c)	EPR	Photothermal therapy	[226]
Breast	Au	32–49 core, 27 spikes	Stars of different core geometry: nanospheres, nanocubes, nanorods	SERS	Subcutaneous 4T1 (BALB/c)	CD44	Photothermal therapy	[227]
Bladder	Au	450	Sphere	PET (^18^F)	Orthotopic MB49 (C57BL/6JRj)	No - intravesical injection	Radiotherapy (^131^I)	[228]
Breast	Au, FeO	160	Janus spherical particles (half-coated with Au)	MRI	Subcutaneous 4T1 (BALB/c)	No–intratumoral injection	Photothermal therapy + chemodynamic therapy	[124]
Breast	Au, Gd3+ Au@Cu2Se	185–214	Nanostar decorated with core-shell nanospheres	SERS, MRI	Orthotopic 4T1 with induced distant metastases in lungs (BALB/c)	EPR	Photothermal therapy + anti-PD-1 immunotherapy	[229]
Glioblastoma	Au	91.1	Star	SERS	Orthotopic GL261 (C57BL/6)	CD47	Photothermal therapy + innate immune checkpoint blockade	[194]
Breast	Au	102 (±4.3) x 16 (±3.8)	Rod	SERS	Subcutaneous 4T1 (BALB/c)	EPR	Photothermal therapy	[230]
Breast	Au	108.5 (±1.5)	Star	SERS	Subcutaneous SKBR-3 (BALB/c-nu)	HER2	Photothermal therapy	[189]
Breast	Au	50	Sphere	SERS	Spontaneous mammary tumor (MMTV-PyMT)	CD44	No	[231]
Lung	Au, Gd(III)	200	Nanocluster assembly	NIR-II photoluminescence	Orthotopic 4T1 (BALB/c)	EPR	No	[216]
Breast	Fe_3_O_4_, Co	60	Sphere	MRI	Orthotopic 4T1 (BALB/c)	EPR	Photodynamic therapy	[232]
Bladder	Au, Ag	106–125	Star	SERS, photoacoustic	Subcutaneous MB49 (C57BL/6-albino)	EPR	Photodynamic therapy	[233]

## Data Availability

No data was used for the research described in the article.
